# E.L., a modern-day Phineas Gage: Revisiting frontal lobe injury

**DOI:** 10.1016/j.lana.2022.100340

**Published:** 2022-08-11

**Authors:** Pedro H.M. de Freitas, Ruy C. Monteiro, Raphael Bertani, Caio M. Perret, Pedro C. Rodrigues, Joana Vicentini, Tagore M. Gonzalez de Morais, Stefano F.A. Rozental, Gustavo F. Galvão, Fabricio de Mattos, Fernando A. Vasconcelos, Ivan S. Dorio, Cintya Y. Hayashi, Jorge R.L. dos Santos, Guilherme L. Werneck, Carla T. Ferreira Tocquer, Claudia Capitão, Luiz C. Hygino da Cruz, Jaan Tulviste, Mario Fiorani, Marcos M. da Silva, Wellingson S. Paiva, Kenneth Podell, Howard J. Federoff, Divyen H. Patel, Fred Lado, Elkhonon Goldberg, Rodolfo Llinás, Michael V.L. Bennett, Renato Rozental

**Affiliations:** a Instituto de Ciências Biomédicas, CCS, Universidade Federal do Rio de Janeiro, RJ, 21941-902, Brazil; b Miguel Couto Municipal Hospital, Rio de Janeiro, RJ, 22430-160, Brazil; c Vassar College, Poughkeepsie, NY, 12604, USA; d Dept Neurocirurgia, HUGG, Universidade Federal do Estado do Rio de Janeiro (UNIRIO), RJ, 20270-004, Brazil; e Dept Neurologia, Universidade do Estado de São Paulo, SP, 05402-000, Brazil; f Pontificia Universidade Catolica do Rio de Janeiro, RJ, 22451-000, Brazil; g Centro de Neurologia da Cognição e do Comportamento Ltda, RJ, 22071-000, Brazil; h Centro Universitario IBMR, RJ, 22631-002, Brazil; i MRI Clinica de Diagnostico por Imagem (CDPI/DASA), Rio de Janeiro, 22271-040, Brazil; j University of Tartu, Institute of Psychology, Tartu, Estonia; k Instituto de Biofísica, Universidade Federal do Rio de Janeiro, RJ, 21941-902, Brazil; l Dept Neurologia, HUCFF, Universidade Federal do Rio de Janeiro, RJ, 21941-902, Brazil; m Neurological Institute, Houston Methodist, TX, 77030, USA; n Georgetown University, Washington, D.C., 20057, USA; o Genome Explorations, Memphis, TN, 38132, USA; p Northwell Health, Manhasset, NY, 11030, USA; q Dept Neurology, New York University, School of Medicine, NY, 10016, USA; r Dept. Physiology and Neuroscience, New York University, School of Medicine, NY, 10016, USA; s Dept Neuroscience, Albert Einstein Coll Medicine, Bronx, NY, 10461, USA; t Centro Desenvolvimento Tecnológico (CDTS), FIOCRUZ, Rio de Janeiro, 21040-361, Brazil

**Keywords:** Traumatic brain injury (TBI), Phineas Gage, Prefrontal cortex (PFC), Corpus callosum (*C.C*.), Magnetic Resonance Imaging (MRI), Neuropsychological tests, Transcranial Magnetic Stimulation, Low Frequency Oscillations, TBI, Traumatic brain injury, C.C., Corpus callosum, TMS, Transcranial magnetic stimulation, PFC, Prefrontal cortex, MRI, Magnetic Resonance Imaging, LFO, Low frequency oscillations (EEG)

## Abstract

**Background:**

How the prefrontal cortex (PFC) recovers its functionality following lesions remains a conundrum. Recent work has uncovered the importance of transient low-frequency oscillatory activity (LFO; < 4 Hz) for the recovery of an injured brain. We aimed to determine whether persistent cortical oscillatory dynamics contribute to brain capability to support ‘normal life’ following injury.

**Methods:**

In this 9-year prospective longitudinal study (08/2012-2021), we collected data from the patient E.L., a modern-day Phineas Gage, who suffered from lesions, impacting 11% of his total brain mass, to his right PFC and supplementary motor area after his skull was transfixed by an iron rod. A systematic evaluation of clinical, electrophysiologic, brain imaging, neuropsychological and behavioural testing were used to clarify the clinical significance of relationship between LFO discharge and executive dysfunctions and compare E.L.´s disorders to that attributed to Gage (1848), a landmark in the history of neurology and neuroscience.

**Findings:**

Selective recruitment of the non-injured left hemisphere during execution of unimanual right-hand movements resulted in the emergence of robust LFO, an EEG-detected marker for disconnection of brain areas, in the damaged right hemisphere. In contrast, recruitment of the damaged right hemisphere during contralateral hand movement, resulted in the co-activation of the left hemisphere and decreased right hemisphere LFO to levels of controls enabling performance, suggesting a target for neuromodulation. Similarly, transcranial magnetic stimulation (TMS), used to create a temporary virtual-lesion over E.L.’s healthy hemisphere, disrupted the modulation of contralateral LFO, disturbing behaviour and impairing executive function tasks. In contrast to Gage, reasoning, planning, working memory, social, sexual and family behaviours eluded clinical inspection by decreasing LFO in the delta frequency range during motor and executive functioning.

**Interpretation:**

Our study suggests that modulation of LFO dynamics is an important mechanism by which PFC accommodates neurological injuries, supporting the reports of Gage´s recovery, and represents an attractive target for therapeutic interventions.

**Funding:**

Fundação de Amparo Pesquisa Rio de Janeiro (FAPERJ), Universidade Federal do Rio de Janeiro (intramural), and Fiocruz/Ministery of Health (INOVA Fiocruz).

**Research in context**

**Evidence before this study**

The historical case of Phineas Gage (1848) is an integral part of medical folklore, illustrating the resilience of the human brain and the involvement of the frontal lobes in problem solving, spontaneity, memory, initiation, judgement, impulse control, and social and sexual behavior. However, the dynamics of damaged frontal lobe continue to be the subject of controversy, and functional recovery has remained a matter of debate since Gage's accident. On a parallel track, sustained high amplitude low-frequency oscillations (LFO) generated in dysfunctional cortical networks has been suggested to have a high correlation with cortical dysfunction and as a marker of cortical disconnection.

**Added value of this study**

Our study suggests that modulation of LFO dynamics is an important mechanism by which PFC accommodates neurological injuries, supporting the reports of Gage´s recovery and represents an attractive target for therapeutic interventions.

**Implications of all the available evidence**

Modulation of LFO dynamics is an important mechanism by which PFC accommodates neurological injuries, representing an attractive new target for therapeutic interventions after frontal lobe injury.

Alt-text: Unlabelled box

## Introduction

Today, some 170 years after the landmark case of Phineas Gage, who exhibited significant changes in personality as a result of frontal lobe damage, the so-called ‘riddle of the frontal lobes’ has yet to be solved.^1^ The characterization that ‘Gage was no longer Gage’,^2^ albeit poorly documented, was consonant with the rise of cortical localizationist theories of brain functioning in the mid-to late 19^th^ century, setting out the context of the ‘localization debate’ for the nascent science of the ‘brain and mind’.^1^ Split-brain studies with sectioned corpus callosum (*CC*), the major neural pathway that connects homologous cortical areas of the cerebral hemispheres, provided evidence for the view of hemispheric asymmetries and specialization^3^ and afforded insights into neural mechanisms. These studies also unveiled areas of the *CC* dedicated to the transfer of visual, somatosensory and motor information, which are stronger for handedness than for footedness.^4^ These ideas bring us to the current view of a language-dominant left hemisphere while visuospatial (e.g., geometric forms) and other features show more lateralization in the right hemisphere.^5^ The dynamics of damaged prefrontal cortices (PFC), however, critical for cognitive, asocial behavior, decision-making functions and moral judgment, continue to be the subject of controversy, and functional recovery has remained a matter of debate since 1848.

White matter (WM) change is central to a range of neurological conditions, including traumatic brain injury (TBI), psychiatric disorders and in healthy aging.^6^, ^7^, ^8^ Consistent with neuropathological findings, neuroimaging and neuropsychological literature has focused on executive functioning decline in subjects who underwent brain signal processing by quantitative MRI measures such as lesion load, demyelination, WM integrity and altered gray matter (GM) volume. At the electroencephalographic level (EEG), sustained high amplitude low-frequency oscillations (LFO) (<4 Hz) generated in dysfunctional cortical networks in TBI patients, psychiatric disorders and aging, has been shown to have a high correlation with neuropsychological assessments in neurodegenerative processes^9^^,^^10^ and it is regarded as a marker of cortical disconnection, leading to deafferentation of networks from their major input source.^11^ Indeed, while an intact thalamus is required for proper brain function during both sleep and wakefulness in controls (CTRL), predisposing to declarative learning, memory^12^ and sleep,^13^ and thus improving cognitive performance, the sufficiency of the cortex for the generation of abnormal slow waves is supported by corticocortical connections following thalamectomy.^14^ In turn, treatment-associated decreases in frontal LFO activity have been suggested to improve functional outcome and impaired attention in mental disorders.^15^ However, no definitive explanation for how LFO interact with other recurring changes in excitability and is modulated in frontal lobe injury was provided so far.

The overarching goal of the current study, prompted by clinical, neuroimaging, neuropsychological and EEG evidence paired with TMS, is to provide clues as to how PFC accommodates to neurological insults and, in particular, to test the unsettled concept that down modulation of abnormal LFO has mechanistic implications for compensation on executive dysfunction. Identifying compensatory responses associated with LFO over the injured hemisphere may facilitate determination of the extent to which neural recruitment in injury represents reorganization or functional engagement of existing latent networks. Finally, while there is no question that, immediately following the accident there were changes in Gage's personality, other reports nevertheless state that Gage actually recovered and resumed something resembling a ‘normal life’ – a possibility that, if substantiated, could impact significantly our understanding of the brain's plasticity and treatment strategy.

## Methods

LFO construct validity was performed by determining correlations among clinical examination, physiological recordings, commonly used imaging technology and neurobehavioral & sensorimotor tests of known frontal lobe vulnerabilities in controls and E.L. On the basis of the definition of multitasking, the group created a list of tasks to be tested on affective, cognitive and behavioral sub-domains. Magnetic stimulation, a technique that allows us to temporarily interfere with brain function, was aimed at proof-of-concept testing.

Because of the large amount of assays employed, materials, the following methods and well-established protocols are described in detail as “Supplementary Methods”: clinical exam, quantitative electroencephalography (qEEG), MRI image acquisition & segmentation (FreeSurfer), post-processing and volume measurements, 3D printed brain models, electrooculography (saccadic and antisacaddic tasks), oxcarbazepine (anticonvulsant) test and a set of neuropsychological and behavioral tests, including “apathy evaluation” and assessment of sexuality (BIQS).

### Matched control sampling

The control sample consisted of consecutive healthy male participants, strictly right-handed, not taking any medications, with a mean age of 21.6 ± 0.9 (20-30 years) (*n* = 5) (qEEG), 30 ± 1.3 years (20-35 years) (*n* = 10) (TMS/ROFC), 33.4 ± 2.4 (20-45 years) (*n* = 10) (Divided Attention), 26.7 ± 2.7 (20-35 years) (*n* = 4) (TMS/EEG/apathy) and 21.6 ± 0.9 (20-30 years) (*n* = 5) (EOG). In general, the participants were all volunteers made aware of the possibility of volunteering by a general notice, who responded to an advert in local recruitment flyers, University hospital flyers on volunteer opportunities or active recruitment approaches to take part in research on “neuropsychological testing”. Volunteers with incomplete junior high school were selected to match E.L. level of education (*Divided Attention*) (Supplementary Methods).

### Procedures

#### Transcranial magnetic stimulation (TMS). Neuronavigation

Patients underwent MRI using a 1.5 T SPREE (Siemens AG, Erlangen, Germany). T1-weighted images were acquired in the sagittal plane with a 3D magnetization prepared rapid acquisition gradient echo sequence (MPRAGE) (Supplementary Methods). The MRI images were recorded in Digital Imaging and Communications in Medicine format (DICOM). A neuronavigational device (BrainSight 2, Rogue Research Inc, Montreéal, QC, Canada) sensor was applied to guide the coil positioning, which allowed visualization of the angle of impact for the magnetic impulse onto the brain surface. Subsequently, a system was introduced that also facilitated elucidation of the exact strength and extent of the induced electrical field, depending on the depth of the area under the coil. For the TMS mapping, the patients were seated on a chair with a headrest. Focal single-pulse TMS was delivered to the motor cortex with a figure-of-eight magnetic stimulator (diameter 70 mm; 9 turns of the wire; peak magnetic − 2.2 T) (MagPro × 100; MagVenture A/S, Farum, Denmark). The magnetic coil was placed with navigation. We performed a co-registration of MRI for face recognition by points (glabella, nasion, right tragus, and left tragus). The motor cortex was identified, using TMS impulses in the cortex to produce movement in the contralateral hand. The motor threshold was considered as the lowest intensity of TMS single pulses required to induce a motor-evoked potential (MEP) with an amplitude ≥50 µV, in five out of ten consecutive trials.

Methods used to record EMG signals of the first dorsal interosseous muscle, to apply repetitive TMS stimulation (rTMS) of the dorsolateral PFC (dlPFC) and left Primary Motor Cortex (left PMC) and continuous theta-burst stimulation (cTBS) of the ventromedial PFC (vmPFC) are described in *Supplementary Methods*.

### Neuropsychological testing

A comprehensive neuropsychological test battery to assess frontal lobe dysfunction was administered, comprising tests covering cognitive function, neuropsychological domain and sexual arousal. We aimed for a battery of tests, including the executive control battery, based on approaches and procedures developed by A. Luria and E. Goldberg, and other gold-standard tests of cognitive assessment, such as Iowa Gambling Task (IGT) (self-account measures), Cognitive Bias Test (CBT) (actor-centered decision making), Wisconsin Card Sorting Test (Nelson version) (WCST) (veridical decision making) and Rey-Osterrieth Complex Test (short- and long-term visuospatial memory).^16^ The neuropsychological profile assessing each group of executive functioning is grouped in Table 1, Extended Table 1 and described in Supplementary Methods.

**Table 1:** E.L.: predicted difficulties and impairments in cognitive function and in neuropsychological domain on the basis of standard neuropsychological measures.

Cognitive function	Tests	Performance
*Verbal & Visuospatial Reasoning*	Wechsler Adult Intelligence Scale (WAIS III)	**N**
*Attention*	Stroop Test (W, C, CW and Interference Index)	deficit **N**
*Visuospatial Memory*	Rey – Osterrieth Complex Figure Test (ROCFT)(immediate and delayed recall)	**N**
*Logical Memory*	WMS3 (immediate and delayed recall), story recall	**N**
*Motor Functions*	Finger tapping test (unimanual)	**N**
*Decision making*	Iowa gambling test	**N**
*Executive Functions*	ROCFT, Wisconsin Card Sorting Test (WCST), Tower of London	**N**
**NEUROPSYCHOLOGICAL DOMAIN**		
*Verbal Fluency (2 min)*	Semantic and phonemic	*borderline*
*Attention/Executive*	Trail Making Test (A, B, B-A)	deficit, **N**, deficit
*Abstract Reasoning*	Raven Progressive Matrices (PM38)	**N**
*Short-Term Auditory-Verbal Memory*	Span Forward and Backward, Immediate Recall	**N**
*Long-Term Auditory-Verbal Memory/Episodic Memory*	CVLT (total learning, delayed recall, recognition)	deficit
*Short- and Long-Term Visuospacial Memory*	Corsi Span	deficit
	RCFT recall (immediate and delayed)	**N**
	DMS-48, Ruche de Violon Test	deficit
*Word Retrieval*	Boston Naming Test (BNT)	**N**
*Visuospacial Abilities*	ROCFT copy	**N**
*Non-Verbal Memory*	Facial Recognition Test (Benton)	**N**
*Verbal Memory*	Grober and Buschke Test: short term and delayed recall recognition	
		deficit **N**
*Mood*	MMSE, BDI	**N**
**SEXUAL AROUSAL**	BIQS	**N**

The executive control battery (ECB) – we included the bimanual (reciprocal) coordination. The test allows the eliciting of various types of motor perseverations, stereotypes, and other deficits of sequential motor organization.^16^

Rey-Osterrieth Complex Figure (ROCF) (visuospatial memory). This test allows assessment of a variety of cognitive processes, including planning, problem-solving strategies, as well as perceptual, motor, and episodic memory functions. Participants were presented with a blank sheet of paper and were asked to make a copy of the figure with their right-hand (dominant hand) as carefully as possible. Subjects were allowed to rotate their drawings, which was aimed at maximizing the quality of their copies but were not allowed to rotate the figure. Erasing was allowed. Each subject delivered the copied figure within 5 min. After 3 min, the subjects were asked to reproduce the design from memory (3 min recall) and then again after a 30 min hiatus (30 min recall). Copy and reproductions of the ROCF were scored using original Scoring System by André Rey.^17^

### Statistics analyses

#### General statistical analysis

Descriptive statistics (mean±SEM) were calculated with Excel (Microsoft) or GraphPad Prism (GraphPad Software). Mean±SEM was used to report statistics unless otherwise indicated. All statistical analyses were conducted using GraphPad Prism. A *P* value <0.05 was considered statistically significant.

### EEG frequency power

Spectral power was averaged at all sensors, the time course of slow wave (SW) activity (0.5–4 Hz) was calculated and the open-source R program with "SingleCaseES" and "scan" packages was used to perform the Non-overlap of all pairs (NAP) method^18^ to evaluate the changes in SW activity associated with the TMS-OFF (resting state) and the TMS-ON blocks within E.L. and CTRL (*n* = 4), 26.7 ± 2.7 years (20-35 years) (*n* = 4). Similar procedures were performed to evaluate SW activity during finger-tapping paradigm. ***dlPFC rTMS Rey-Osterrieth Complex Figure assay.*** E.L. single-case data analysis and comparison to a CTRL sample, 30 ± 1.3 years (20-35 years) (*n* = 10) (TMS), were performed with the open source software Singlims ES using the Crawford and Howell's and the Crawford & Garthwaite´s methods,^19^ supplemented by point and interval estimates of effect sizes tests if E.L's score was significantly below CTRL. This methodology provides a point estimate and percentage of the abnormality of the score, with the use of *P* value, and sets confidence limits on the abnormality of a patient's score using non-central t-distributions. CTRL sample sizes were chosen based on prior standards established in previous published studies in neuropsychology field to achieve 90% power in a one-tail test with α=0.05 and Z-CC ≥ 4.^19^

#### Ethics committee

The present study was in strict accordance with the NIH Guide involving Human Subjects and approved by Institutional Ethics Committees (CAAE 96263418.0.0000.5279 – CONEP/Plataforma Brasil).

### Role of the funding source

The sponsors of this study had no role in study design, data collection, data analysis, data interpretation, or writing of the report. R. Rozental, M.V.L. Bennett and P.H.M. de Freitas had full access to all the data in the study and all authors had final responsibility for the decision to submit for publication.

## Results

### History and physical examination: ‘replicating gage’

**Figure 1:**
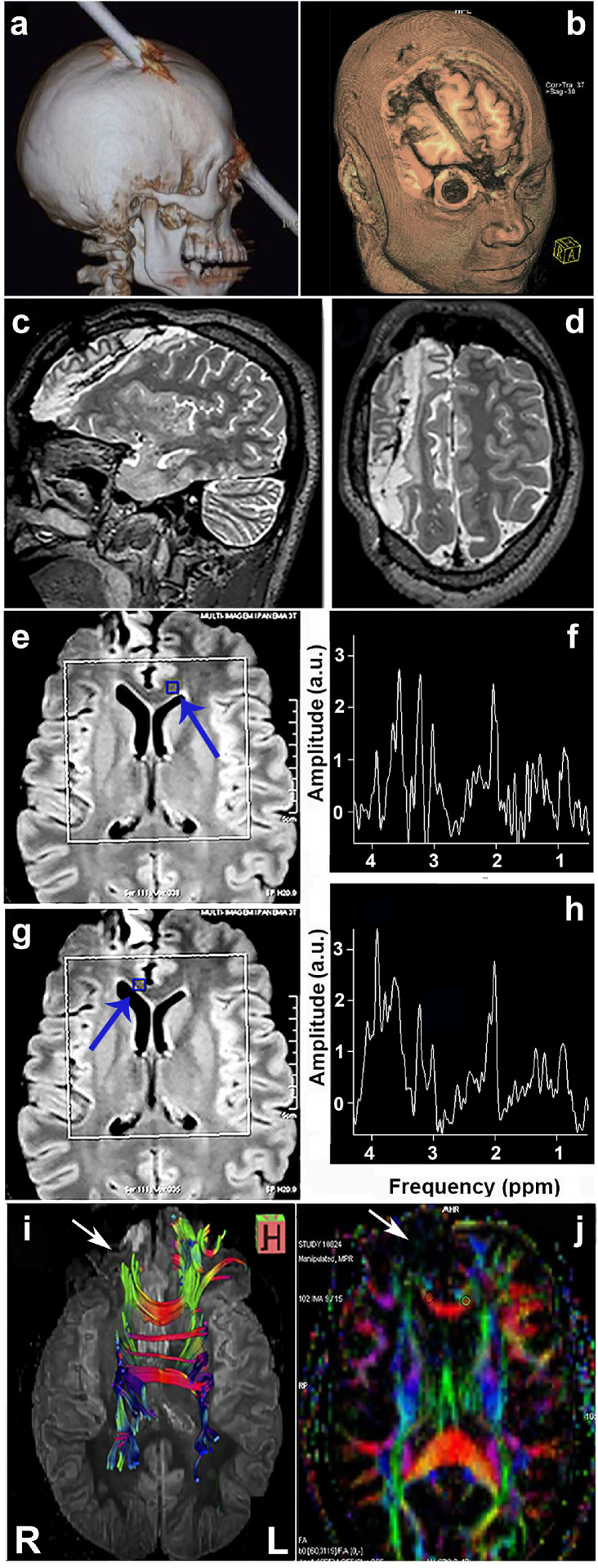
Path of the iron bar through E.L.’s skull and its effects on white matter structure. (a) Lateral view of the transfixed skull with an iron bar on the CT scan reconstruction. (b) T1 weighted MRI reconstruction revealed the trajectory of the iron bar. Lateral (c) and transverse (d) sections (T2-weighted MRI images), 18 months after the accident, suggestive of a right frontal lobe disconnection; (e,g) FLAIR MRI spectroscopy (single-voxel) was applied to regions of interest (blue squares), indicated by blue arrows, on the anterior *corpus callosum (CC).* (f,h) An expected decrease in spectrum of resonances in N-acetyl-aspartate (NAA) (2ppm), marker of neuronal integrity, is illustrated. (i) Axial view of a tractography suggesting a difference in the volume of the association fiber tracts that connect temporo-parietal cortical regions with right frontal lobe (white arrow). (j) Fractional anisotropy (FA) map, a diffusion tensor imaging technique, revealed that the injured area was associated with greater anisotropy reduction in frontal right brain regions (white arrow). R (right hemisphere), L (left hemisphere).

History repeated itself in August of 2012 (Gage vs E.L., Supplementary Clinical Case History). In 1848, Phineas Gage, a 25-year-old American construction foreman, sustained extensive frontal lobe damage after an iron bar - 31 mm in diameter, 1.06 meters long and weighing 6.1 kg - was propelled through the front part of his head. Gage reportedly experienced pronounced personality changes due to lesions from this accident that are presumed to have involved the left frontal region. 164 years later, at 9:30 am on August 15, 2012, E.L., a 24-year-old Brazilian man, who was kneeling on the ground floor at a construction site, experienced a similar accident; a round steel bar - 16 mm in diameter, 2.56 meters long and weighing 4 kg – fell from the 5th floor (15 m height), impaling his skull obliquely (superior-anterior) on the right side at an estimated velocity, at contact, of 17.32 m/sec (62.35 km/hr). The entry-point was about 1.5 cm posterolateral to bregma and the exit-path of the rod made contact with the glabella (Figure 1a,b, Extended Data Figure 1a,d,e). E.L. was discharged from hospital care on August 30th, 2012 and returned to work on a construction site in November, 2012 (Supplementary Clinical Case History). E.L. continued functioning normally for approximately 9 months after his return to work, until he experienced a tonic-clonic seizure. After the seizure, he stopped working, and began treatment with oxcarbazepine (OXC) (300 mg at 8-hr intervals), an anticonvulsant (AED). In the 94 months since beginning treatment with OXC, he experienced motor seizures 11 times (72% within the first 23 months of treatment), all lasting less than 1 min. Symptoms started on his left arm, while E.L. was fully aware of the surroundings, before losing consciousness and spreading to involve both sides of his body. All episodes, except for the first one, were due to medication noncompliance (Supplementary Clinical Case History).

### Medical history (2012–2020)

E.L.'s medical history is otherwise unremarkable. E.L., who is right-handed, suffered traumatic injury (TBI) of the right frontal lobe, and now experiences post-traumatic epilepsy as his only notable symptom. Neurological examination shows only mild abnormalities: right-sided hyposmia as result of direct damage to the nose and sinus, injury to the anterior cranial fossa, and frontal lobe involvement. He exhibits subtle asymmetry of reflexes between left and right arms, mild incoordination with rapid alternating movements in the left hand, and difficulty maintaining left gaze, which is consistent with a lesion to the right frontal lobe including the right frontal eye fields, and evidence suggesting a grasp reflex in the left foot, a frontal release sign. Coordination testing showed normal symmetrical movements without dysmetria, or incoordination, on finger to nose testing, normal forearm and finger rolling tests using rapidly alternating movement, and on sequential active finger-to-thumb tapping, rapidly touching the thumb to each fingertip, during unimanual movements with either the left or right hand (Extended Data Figure 3) (Supplementary Clinical Case History - Neurological Examination). However, there was incoordination of sequential finger-to-thumb tapping movements in the left hand during bimanual movements suggestive of failure of compensatory mechanisms during multitasking (Supplementary Movie 1) (Supplementary Clinical Case History).

### Functional magnetic resonance imaging (fMRI) – extent of cortical gray matter (GM) and white matter (WM) damage

A set of 5 MRI data collection from E.L. over the years, was used for this study. Cross-sectional, parasagittal and longitudinal MRI image scans (2012, 2013, 2014a,b and 2020) revealed an extensive right unilateral frontal lobe lesion affecting orbital, polar and anterior regions of the PFC. Figure 1b-e,g, Extended Data Figure 1d-e,g-h, Extended Data Figure 2 illustrate the affected areas, involving the orbital frontal cortex (Brodmann´s cytoarchitectonic fields 11 and 12), the polar and anterior mesial frontal cortices (fields 8, 9, 10 and 32), the dorsolateral field 46 and the mesial aspect of field 6 (the supplementary [SMA] and the premotor [pre-SMA] areas). MRI scans over time have not detected enlarging lesions, atrophy or damage outside of the frontal lobe. The amount of right global hemisphere volume loss due to the iron bar was 11.2%, affecting regionally specific gray matter volume (4.6%), cerebrospinal fluid (CSF) and white matter (WM) volumes (6.6%), matching estimations of GM (up to 4%) and of WM (up to 11%) reported in Gage´s computational lesion studies. E.L. total intracranial volume (TIV) was 1,500.4 ml compared to the mean ± SEM CTRL 1,419.4 (23.7) ml (*n* = 16 patients), and his estimated left lateral ventricle volume (LVV) was 56.6 ml compared to 64 ml of his contralateral one (right ventricle). Findings of hippocampal sclerosis were not suggested by long-term MRI scans (07/2020) (Figure 5d). Visualization of fully reconstructed E.L.’s 3D brain model in polyamide helped examine the extent of both cortical and subcortical lesions in close detail (Extended Data Figure 4) (Supplementary Movie 2) (Supplementary Methods).

Connectivity was damaged between the right PFC and other major lobes of the right hemisphere, in addition to the left frontal lobe. WM lack of integrity was identified in the following: superior longitudinal fasciculus (SLF), which connects bi-directionally the parietal, occipital and temporal lobes with ipsilateral frontal cortices; superior fronto-occipital fasciculus (SFOF), which connects frontal and occipital lobes; and, cingulum fasciculus (CF), which courses behind the splenium up to the frontal lobe, extending longitudinally above the *C.C.* - (Figure 1i,j; Extended Data Figure 5). Quantitative Diffusion Tensor Imaging (DTI) did not detect gross changes in volumetric imaging (total and regional) in E.L.’s *C.C.*, despite the nature of his frontal injury. There were no significant differences in midsagittal measures of anterior-posterior extent of *C.C.* between E.L. and CTRL (*n* = 16), even though E.L.’s anterior segment (760.5 mm^3^) was thinner while his central-posterior (453.8 mm^3^ [mid-ant]; 469.6 mm^3^ [central]; 483.6 mm^3^ [mid-post]; and 878.2 mm^3^ [post]) segments were thicker than the average for the matched CTRL (*n* = 16)(868.3 ± 32.68 mm^3^ [ant]; 433.6 ± 19.2 mm^3^ [mid-ant]; 429.9 ± 18.43 mm^3^ [central]; 397.3 ± 19.59 mm^3^ [mid-post]; 991.2 ± 41.02 mm^3^ [post]) (Supplementary Movie 3). The groups did not differ in total *C.C.* volume (3,045.6 mm^3^ [E.L.] vs 3,120.3 ± 94.13 [matched-CTRL]).

### Assessment of functional tasks performance and sexuality after right frontal lobe damage

E.L. and CTRL ratings on a measure of frontal cognitive, neuropsychological and sexual behaviour were assessed using standardized and gold-standard neuropsychological battery tests. Table 1 and Extended Table 1 display the abilities assessed by specific testing, including reading, language usage, attention, learning, processing speed, reasoning, remembering, problem-solving, mood and personality and more. No apparent direct relationship has been found between cognition, executive function or behavioural impairment in the clinical course of his injury during the last 9 years.

E.L. executive function and behaviour, for the most part, did not deteriorate since his accident. E.L. has not expressed neuropsychological problems in family, sexual, professional and social life and did not present attentional dysfunction. In general, he performed as well as CTRL in standard tests to assess TBI subjects, among them the WAIS-III (intelligence), Tower of London (planning ability), Boston Naming Test (BNT) (confrontation naming), the Wechsler Memory Scale (WMS) (auditory and visual memory; immediate and delayed memory), Rey-Osterrieth Complex Figure Test (RCFT) (visuospatial perception, skills and recognition memory), Mini-Mental State Examination (MMSE) (cognitive impairment), Beck Depression Inventory (BDI) (depression), Prospective Memory (PM) and tests to evaluate Divided Attention (Table 1; Extended Table 1). Similarly, there was no detectable impairment by gold standard tests of cognitive assessment, such as, Iowa Gambling Task (IGT) (self-account measures), Cognitive Bias Test (CBT) (actor-centred decision making) and Wisconsin Card Sorting Test (Nelson version) (WCST) (veridical decision making). E.L. did, however, show deficits on episodic memory category (Ruche de Violon and Grober and Buschke Tests), visual memory (DMS-48), working memory (Corsi Blocks), color and word cognitive flexibility (Stroop) and attention and task switching (Trail Making Test) (Table 1; Extended Table 1). Among them, on every subtest of the Wechsler Adult Intelligence Scale III (WAIS-III) and on the Divided Attention Test (DAT), tasks commonly thought to be sensitive to the integrity of the frontal cortex, E.L. showed abilities that were either average or superior to the averages for CTRL: 12 (WAIS), 88% (calculation task - DAT) and 17 (cancellation task – DAT). The results of the Wisconsin Card Sorting Test (WCST), the Stroop Colour Interference Test, the Iowa Gambling Test (Extended Data Figure 6) and the Cognitive Bias Task (CBT), regarded as the gold standards of neuropsychological frontal-lobe assessment in patients with lateralized lesions, were also favourable (Table 1; Extended Table 1)

E.L. results were also within normal ranges in the assessment of sexual and behavioural functioning (described hereinafter) (Sexuality questionnaire – Extended Data Figure 7 a,b). We also did not learn of any problems related to higher brain dysfunction in E.L.’s family and/or social lives after his discharge from the hospital. The association of AED and abnormal slow electrographic activity with some cognitive deficits, however, cannot be ruled out (discussed hereinafter). E.L. returned to work with the same construction company and was able to handle a workload comparable to before his accident. There were no noticeable declines in his mental processing, moral reasoning, social behaviour, capacity to solve daily problems, ability to interact with his co-workers or ability to act efficiently (Social Interaction - Supplementary Clinical Case History).

**Figure 2:**
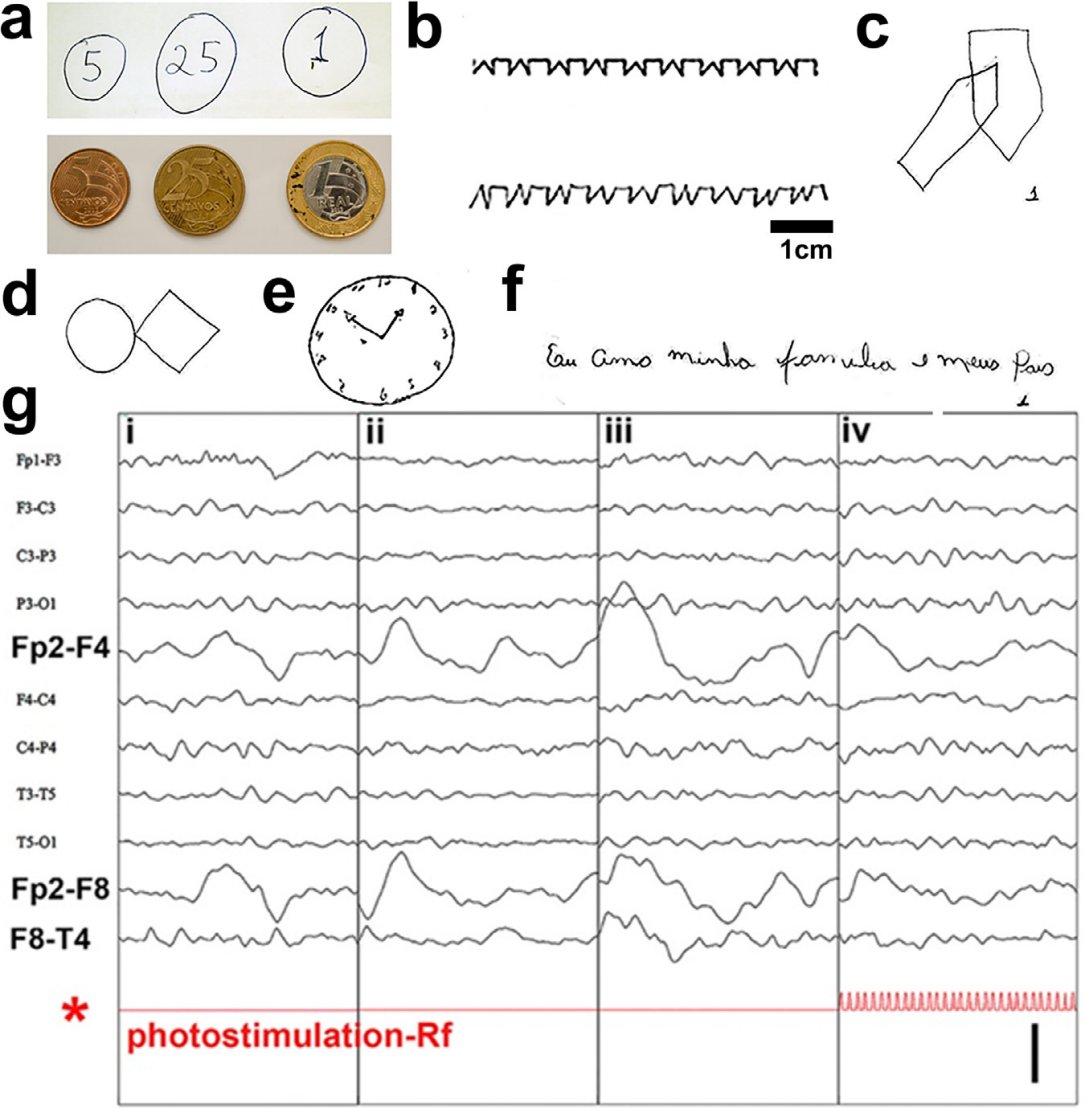
E.L.: Figural fluency, emotional behaviour and EEG as a measure of right frontal lobe injury. This battery of tests was aimed at identifying dysfunction, such as attention and concentration, self-monitoring, personality, inhibition of behaviour and emotions, and with speaking or using expressive language. (a-d) Representative illustrations of memory for geometric designs, shapes, features and directional orientation. (a, *top*) E.L. was asked to drawn R$ 5 cents, 25 cents and 1 Real coins from memory (i.e., draw-to-command). Coin measurements were compared to the respective official coins (a, *bottom*). (e) The clock-drawing test did not confirm a diagnosis of cognitive deficits in E.L. Assessment focused on size of the clock, graphic difficulties, stimulus-bound response, conceptual deficit, spatial/planning deficit and perseveration. (f) context-dependence of emotions within text, illustrated by “Eu amo minha familia e meus pais” (“*I love my family and my parents*”). (g) Spontaneous EEG (four non-consecutive sec of the same recording session placed side by side). (i) Eye closure sensitivity: the awake EEG is characterized by a posterior dominant alpha rhythm (9-10Hz) and moderate voltage reactive to eye opening and closure. (ii) Awake, eyes-closed resting condition: there is frequent delta slowing seen in the right frontal region (Fp2-F4 and Fp2-F8). (iii) Hyperventilation (HV): HV showed a tendency to increase the focal slowing noted above. (iv) Photic Stimulation (PS): intermittent PS did not alter the background. Calibration bar: 100 µV.

In single task drawings, the phenomenon of spatial hemi-inattention, more common after right than after left hemisphere injuries, tends to be reflected in accuracy (e.g., size, shape, placement on the page) and in the omission of details on the left side of the drawing. Since spatial neglect is a complex disorder, subjects were also required to process information, to extrapolate and to think ahead using working memory in the PFC.^20^ Within this context, no differences were found, between E.L. and normalized test data (Figure 2a-f) (Table 1; Extended Table 1). However, univariate analyses suggested significant differences on a few measures involving working memory. E.L.’s attentional dysfunction was not identified and he performed within the upper range of CTRL (**n = 10)**, in terms of both speed and drawing accuracy (placement, geometric forms and size), demonstrating: i) judgement of line length and orientation; ii) width of angles; iii) positioning of dots into geometrical figures; iv) pinpointing the location of a numeral in geometrical figures; v) mentally rotating figures; vi) recognition of nonsense shapes; vii) determining the number of cubes composing a complex 3D shape; and viii) mentally retrieving and reconstructing images. E.L.’s performance in the ‘clock drawing’ and in the Rey-Osterrieth Complex Figure (ROCF) (copy condition, immediate recall and delayed recall) tests, the most frequently used to assess nonverbal memory function, whose performance, needed for quotidian activities, was ranked in the top 10% of those elaborated among all subjects tested (Figure 5j **E.L. TMS-untreated**).

### Saccadic and antisaccadic tasks

E.L. and CTRL group, 21.6 ± 0.9 (20-30 years) (*n* = 5), composed of male subjects of a similar age as E.L., performed saccadic and antisaccadic tasks under gap and overlap paradigms being quantified latencies of eye movements and tasks error (see Supplementary Methods and Extended Figure 8a-b and Extended Figure 9a-c). E.L. showed difference in prosaccadic and antisaccadic errors to single targets (Extended Figure 8c,d,e,f and Extended Figure 9). Similar trends, however, were not observed when the same CTRL group under oxcarbazepine-influence (OXC-CTRL) (*n* = 5), compared to E.L. Accordingly, the OXC-treated group did not show difference in symmetry (right vs left) of prosaccadic-antisaccadic latency and velocity (Extended Data Figure 8) (Supplementary Methods).

### Functional magnetic resonance imaging (fMRI) – *dual tasks*

Neurological (bimanual finger taps) test proved to be a valuable tool for unmasking functional limitations under dual tasks and paved the way for a strategic management of E.L.

**Figure 3:**
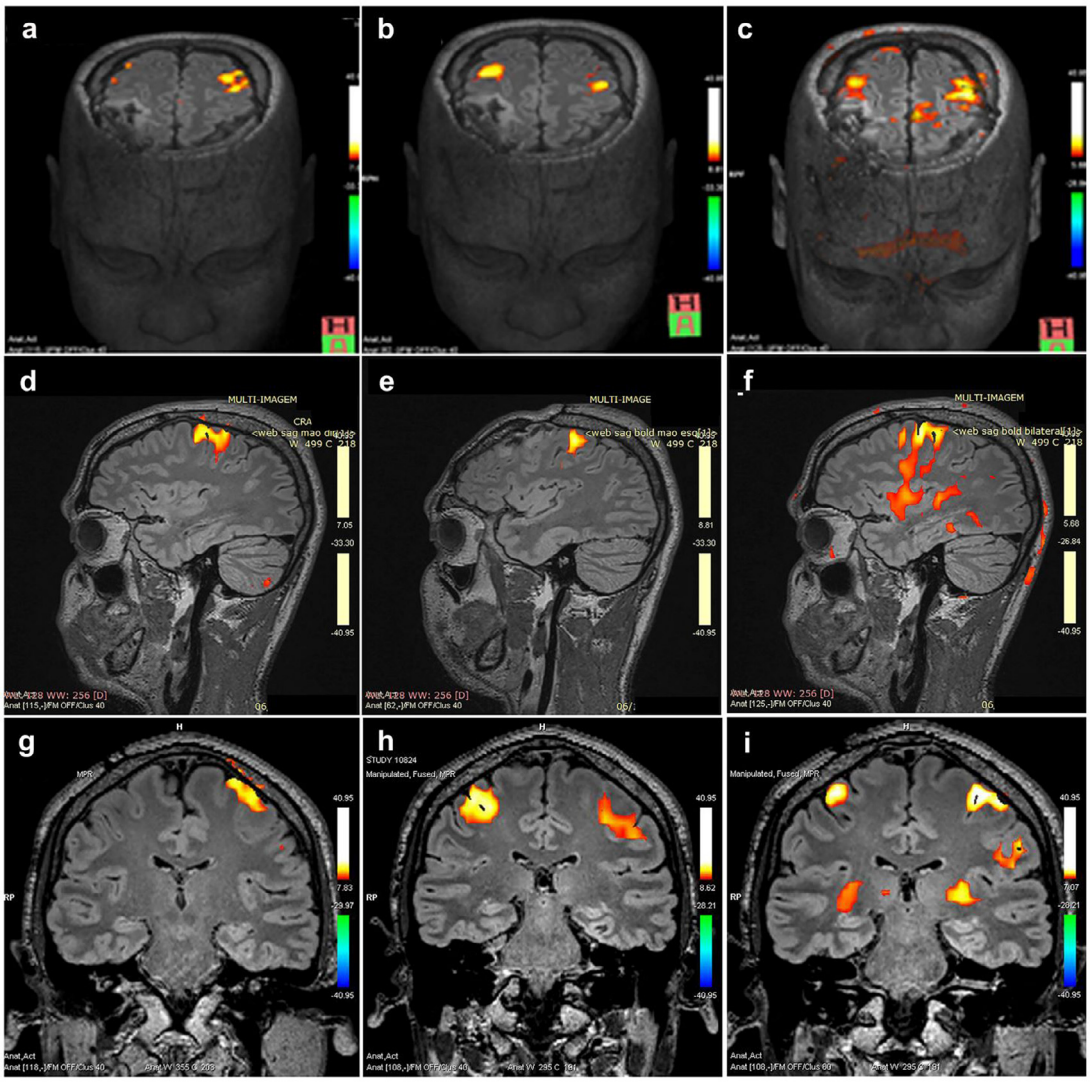
Functional BOLD MRI images (fMRI) of E.L.’s brain activation during sequential finger-to-thumb tapping movements using one hand alone or two hands simultaneously. Axial (a-c), sagittal (d-f) and coronal sections (g-i) offer the possibility of directly investigating cortical brain activation and connectivity during task performance using the right hand alone (a,d,g), left hand alone (b,e,h) or during bimanual movements (c,f,i). Of note, use of either the left hand alone or simultaneously both hands showed a similar trend of activation in the bilateral frontal and parietal regions, known to be involved in working memory. By contrast, for the right-hand alone movements, activation of the motor control regions was lateralized to the left hemisphere. Scale: Z score ranging from -40.95 to 40.95.

On the day of E.L. scanning, the maximum number of index finger-to-thumb taps in 60 sec was obtained for both hands (unimanual movements). The mean number of finger taps for the left and right tasks were 40.7 ± 3 and 41 ± 2,5, respectively. During unilateral tasks, no detectable movements of the contralateral hand, i.e., mirror movements, occurred. Since E.L. was commanded to start the task, and required no decision making on his part, he was less likely to engage motor selection networks that show a bias towards left hemisphere activation.^21^ In general, finger-tapping tasks activated the motor and premotor regions, including the primary motor (M1), ventral premotor (vPM) and dorsal premotor (dPM) cortex (Figure 3a-i). This task was performed with the right hand only, left hand only, and both hands simultaneously (Extended Data Figure 3c-f).

For the right-hand finger-tapping condition, there was robust contralateral (i.e., left hemisphere) activation in the supplementary motor area (SMA), overlying the primary motor (M1), as well as the ipsilateral cerebellum (i.e., right side). In contrast, during unilateral left hand tapping, there was robust bilateral (i.e., both hemispheres) activation in the SMA, M1, vPM and dPM and in the cerebellar hemisphere ipsilateral to the movements. Similar imagery activation patterns were observed for bilateral tapping, dPM activation extending superiorly to the dorsal convexity and vPM activation extending along the precentral gyrus (Figure 3a-i).

### Characteristic of spontaneous EEG

**Figure 4:**
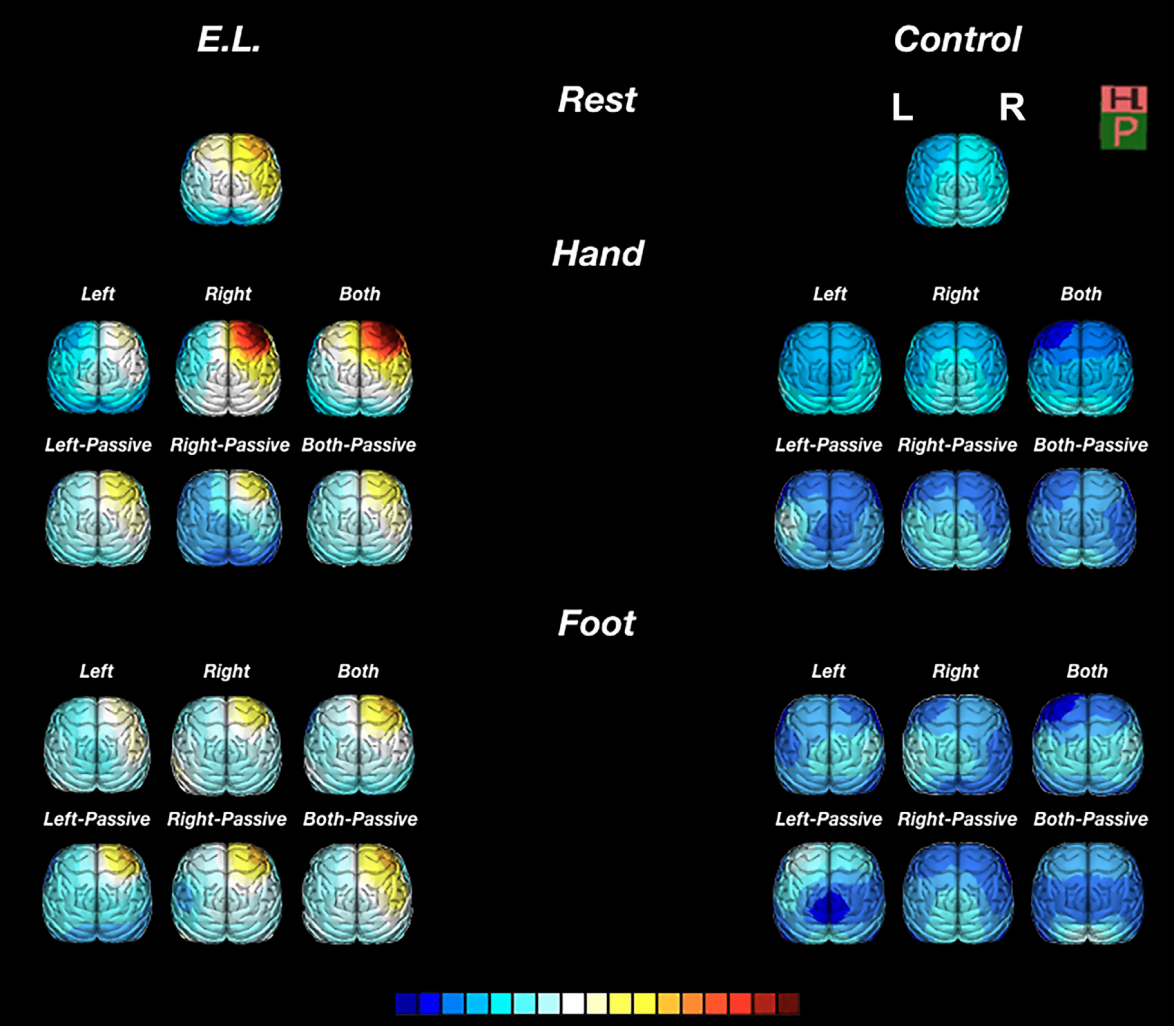
Changes in slow wave amplitude of the quantitative EEG during active and passive (assisted) symmetric movement tasks. The topographic delta frequency band distribution in E.L. and CTRL (resting testing) (top row), during unimanual left/right and bimanual tasks (middle row) and during foot movements (bottom row) is illustrated. Significant differences from baseline for absolute amplitude (µV) in delta frequency were found only in E.L. right frontal region (Fp2) while performing active finger-to-thumb tapping movements – *P* < 0.05 baseline vs. left hand and *P* < 0.05 baseline vs. right hand. Changes in delta wave amplitude during active/passive hand or foot movements were neither documented for CTRL nor for E.L. during either active/passive foot and passive hand movements. Calibration (colour scale): 15-102 µV.

A set of 5 EEG data collected from E.L. over the years (2014; 2015a,b; 2019; 2020) was used for this study. Each epoch was reviewed in two formats: raw EEG and quantitative EEG (qEEG)-only, in which the raw signal is converted into a digital colour form using derived measures. E.L.´s awake EEG at rest is characterized in all EEG datasets by increased asymmetrical right anterior slow dominant rhythm (delta and theta rhythms) of ≤ 4 Hz and of 4-7 Hz and by a symmetrical posterior alpha rhythm of 9-10 Hz, moderate voltage that is reactive to eye opening and closure. Low amplitude beta frequency activity and alpha waves were also present over the anterior head regions, predominantly in the right front central region. During drowsiness, there was attenuation of the awake background and an increase in background slowing. Hyperventilation showed a tendency to increase the focal slowing noted above. Photic stimulation did not alter the background (Figure 2g). A lateralized antero-to-posterior EEG spectral gradient (APSG), which had greater low frequency power from the right prefrontal, frontal lobe and occipital areas, emerged as the most prominent feature at rest associated with E.L. associated brain lesion. The APSG crossed over the genu (in 3 out of 4 runs) and body (in 4 out of 4 runs) of the C.C. to reach the contralateral hemisphere (Figure 2g; Figure 4).

### qEEG signals related to left/right hands and foot movements

Following execution of left-hand motor tasks, repeated measures of frequency of finger-tapping with the right hand alone showed no difference between E.L. and CTRLS (Figure 4) (Supplementary Clinical Case History). In contrast, in the right PFC, delta waves outcome showed a significant increase in frequency to 104.4 ± 1.5 (µV)/epoch (*n* = 3 assays) (*P* < 0.05), while alpha, beta and theta values did not change during the execution of the motor task. A similar motor outcome by E.L. was evidenced during active dual-task performance executed simultaneously with the right- and left-hands (Figure 4). However, during bimanual movements, the mean number of finger taps^−1^ dropped to 29 ± 3, achieving over 32% reduction as opposed to CTRL. In contrast, right foot and left foot alone or simultaneous bipedal movements executed by all subjects, passive or active, independent of the starting foot, did not change the frequency of EEG waves nor did it result in any change in the latency to start performing the task (Figure 4).

### Modulation of cortical network SW oscillatory dynamics by navigated transcranial magnetic stimulation (nTMS)

#### Mapping characteristics

TMS was performed in E.L. and in CTRL (*n* = 4), who were right-handed (age range: 30-35 years). Left Primary Motor Cortex (PMC), dlPFC and vmPFC were focally stimulated sequentially, during three consecutive days in all participants without technical problems or adverse events. Neuropsychological and behavioral performance were determined prior, during and after application of TMS protocols. TMS protocols applied directly on the right hemisphere were not considered because of the nature of E.L.’s frontal injury.

An MRI-guided navigation system was used to estimate in real time an accurate coil positioning on the surface of the skull of the subjects (*see Methods*). Optimal TMS stimulation parameters for modulating non-invasively brain SW activity, identified in the context of the motor system in left PMC (M1) (*day 1*), were subsequently applied to higher level functions such as working memory (*day 2*) and apathy behavior (*day 3*), respectively, in the block design protocol applied in dlPFC and in vmPFC. Participants did not self-report any side effects of stimulation protocols, either excitatory (PMC) or inhibitory (PMC, dlPFC, vmPFC). No apparent abnormal seizure-like activity was detected on EEG recordings following TMS protocols.

#### Threshold of motor evoked potentials (MEPs) and left PMC repetitive transcranial magnetic stimulation (rTMS)

**Figure 5:**
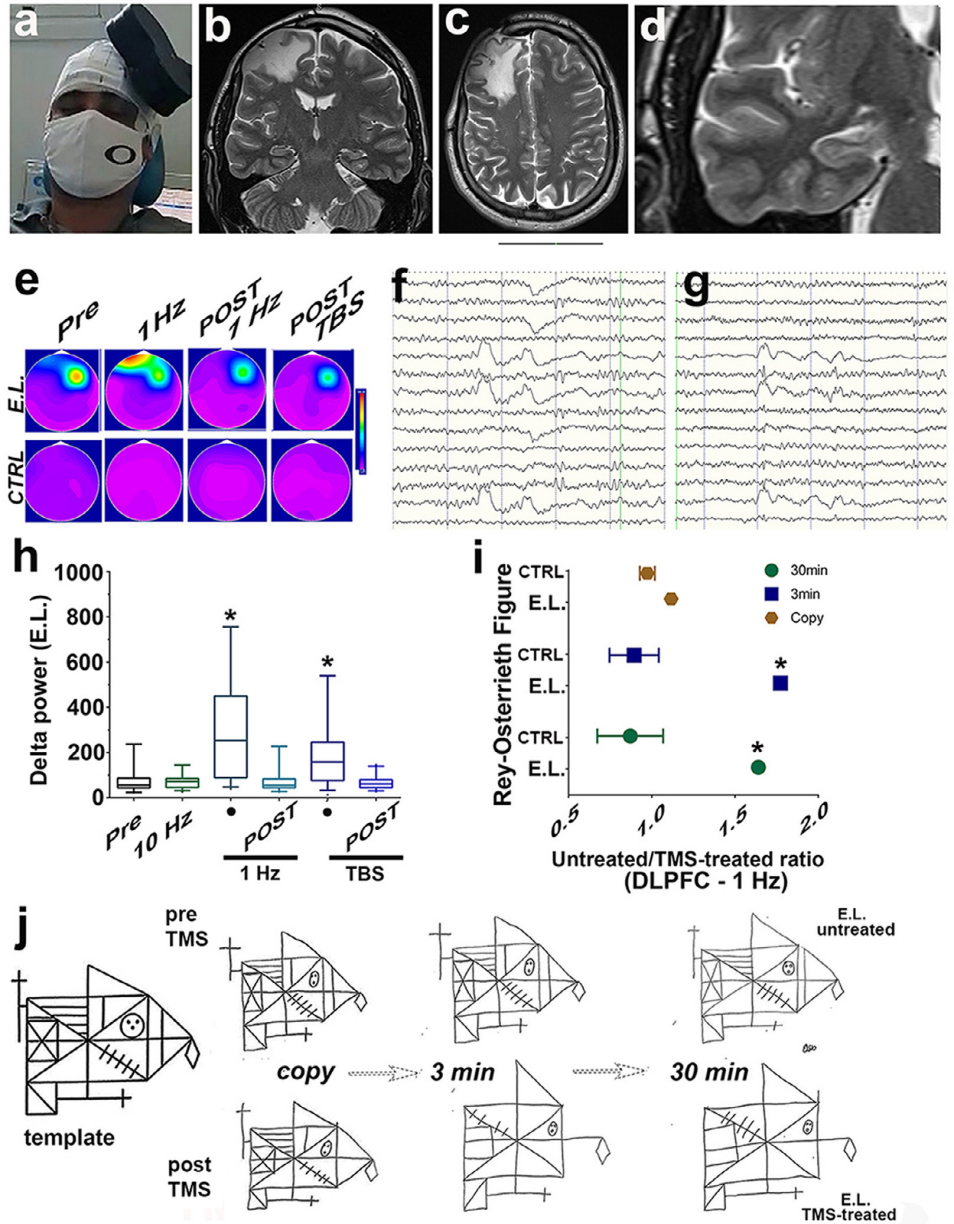
rTMS influences short-term memory and behaviour in E.L., but not in CTRL, by modulating the frequency of slow waves. (a) depicts the position of the conventional TMS figure-of-eight coil with respect to E.L.’s scalp, over the left motor cortex (M1). (b-d) T2- weighted imaging of E.L.’s brain from 2020, 8 years since injury. (b,d) TBI did not result in progressive loss of E.L.’s brain tissue volume. Mesial temporal sclerosis was not detected with regard to hippocampal size and shape, fissure visualization and signal intensity (isotense) to cortical gray matter. (e-h) rTMS-EEG responses to stimulation in E.L. and in CTRL. (e) Topographic plots of the TMS-evoked responses. The extent to which increase in frequency and amplitude of SWs are higher contralateral than ipsilateral to the side of stimulation is illustrated by a train of rTMS at a frequency of 1 Hz (top panel). After rTMS, EEG SW responses were potentiated up to 40 min post 1 Hz (f,h) and up to 60 min post-Theta-burst stimulation (TBS) (g,h), followed by a complete return to background activity (e,h – top panel). (i,j) Subjects were asked to copy the Rey–Osterrieth figure (bottom left – ‘ template’), and then to reproduce it from memory 3–30 min later, prior and after TMS-treatment (1 Hz) applied to the DLPFC. The two groups drawing scores significantly differ between the retrieval rounds at 3 min (* *P* < 0.001) and at 30 min (* *P* < 0.001). rTMS scale (e): 0–500 µV².

Resting- and active motor-thresholds were predetermined in all participants at about 50 µV and 200 µV in the left PMC, respectively, to produce liminal MEP at rest and during isometric contraction of hand muscles (in 50% of 10 trials –*Supplementary Methods*). rTMS inhibitory protocol (1Hz) in left PMC induced high amplitude (> 80 µV) SWs, mostly detected on E.L.’s right-sided scalp (Figure 5e-g), contrasted sharply with the lower amplitude SW of the pre-TMS EEG. Lateralized SWs revealed an antero-to-posterior gradient along the right-sided scalp and, simultaneously, a rapid lateral spread to the left frontal region (Figure 5e). Moreover, in the right PFC, delta slow waves showed a significant increase in frequency and power to 284.56 ± 39.09 µV²/epoch (*n* = 30) (*P* < 0.001) (10 highest epoch: 532.08 ± 46.96 µV²/epoch), maintaining its power for at least 40min (total recording time = 60min). In the subsequent period, with the virtual lesion resolution (*see* Methods), there was a decrease in delta power, with no significant difference (*P* >0.05) from the resting state (73.86± 10.96 event µV²/epoch (*n* = 30) - 10 highest epoch: 127.95 ± 25.51 µV²/epoch). Thus, cross-hemispheric high amplitude SW frontal propagation may rely on E.L.’s anterior *C.C*., albeit expected but not evidently compromised functional integrity of this interhemispheric WM tract. Also, rTMS excitatory protocol (10Hz) in left PMC did not increase the SWs in E.L. (*P* >0,05) 72.54 ± 5.58 µV²/epoch (*n* = 30) (10 highest epoch: 105.3 ± 8.23 µV²/epoch) in comparison to the resting state – TMS-untreated 76.26 ± 10.15 event µV²/epoch (*n* = 30) (10 highest epoch: 134.1 ± 20.21 µV²/epoch) (Figure 5e,h). The triggering of SWs in E.L. could be obtained reliably using inhibitory, not excitatory (10Hz), stimulating conditions at frequency of 1 Hz. In contrast, a similar protocol did not evoke SWs in the CTRL (*n* = 4) (Figure 5e,h), regardless of stimulation site.

#### Left dlPFC contribution to E.L.’s working memory

Here, inhibitory TMS pulses (1 Hz) were used to create a ‘temporary lesion’ of a targeted cortical region, thereby disrupting dlPFC-mediated balance in visuospatial, perception, learning and short-term memory (retention) performance. E.L. apparent deficits on working memory, tailored the selection of the Rey-Osterrieth Figure (ROFC), one of the most frequently used tests for assessing executive dysfunction, to assess the TMS-treated participants. To preclude the possibility that impaired performance could be secondary to rTMS-mediated potential ‘loss of interest’ or ‘apathy’ (*see* below), the intervention using dlPFC stimulation procedure was administered one day prior to that of vmPFC.

Prior to TMS treatment, no differences (*P* >0,05) were found between E.L. and the CTRL (*n* = 10) in the accuracy of the Rey-Osterrieth figural copy (5 min time limit set for the copy), in the immediate recall (IR) (3 min) (and in the delayed recall (DR) (30 min), indicated by Crawford & Garthwaite´s method for detecting the abnormality of a patient´s score. Details are illustrated in Figure 5i,j. However, following TMS treatment, there was a significant difference between E.L. TMS-untreated/treated ratio in comparison with CTRL´s TMS-untreated/treated ratio at the IR (*P* < 0.001 - E.L. 1.756 vs CTL 0.893 [0.02] - Effect Size (Z-CC) 10.128, as well as for the DR (*P* < 0.001 - E.L. 1.619 vs CTL 0.877 [0.03] – Z-CC 6.523) (Figure 5i,j). TMS-treated E.L. reproduced the figure at both measurement points with fewer details and misplacement features. No TMS-mediated decreases in accuracy scores appeared for CTRL regarding IR and DR (mean score of 28.25 [2.06] vs 31.8 [2.18]), and of 28.6 [1.98] vs 32.3 [1.43], respectively).

### rTMS-mediated left vmPFC triggering of slow waves and apathy is state-dependent

Here, a second inhibitory protocol of continuous Theta-Burst Stimulation (cTBS) was applied in left vmPFC, in which three 50-Hz pulses were applied at 5 Hz for 40 sec. E.L. presented increased frequency and power of SWs along with reduction of ‘goal-directed behavior’ (apathy) immediately following cTBS (*P* < 0.001). In support of our main hypothesis following TBS applied to left vmPFC, neither SWs evoked in E.L.’s right hemisphere nor apathy were recorded in or manifested by CTRL. Figure 5 illustrates the results. Both CTRL and E.L. were previously assessed AES-C rating scale suggesting no apathy or apathy related symptoms. For CTRL, no SW pattern or significant behavioral changes (Scale for Assessment of Negative Symptoms (SANS) of 0 [*n* = 4]), emerged following TBS-treatment. By contrast, for E.L., SANS changed from 0 to 45 units (range: 0-125 units), with the presentation of apathy related signs and symptoms 3-5 min after application of TBS protocol, including signs of affective flattening, poverty and increased latency of speech and inattentiveness, paralleling the increased frequency in SWs, and remained for up to 60 min. Return to basal behavioral state was paralleled by return of SWs to pre-TBS stimulation level, determined by EEG recordings (Figure 5e-h). Moreover, in the right PFC, there was a sustained increase in SWs to 184.45 ± 25.75 event (µV2)/epoch (*n* = 30) (*P* < 0.001) (10 highest epoch: 328.35 ± 46.99), maintaining its power for at least 60min (total recording time = 90min). In the subsequent period, with the virtual lesion resolution (*see* Methods), there was a decrease in delta power, with no significant difference (*P* >0.05) from the resting state (68.08± 5.68 event (µV2)/epoch (*n* = 30) - 10 highest epoch: 103.41 ± 8.28).

## Discussion

We have documented the performance of a patient (E.L), a modern-day Gage, on tests of behavioural and executive functions, which were given during a period of 8 years. His severe transfixing frontal lobe damage, remarkably similar to that of Phineas Gage (1848)^22^ (Supplementary Clinical Case), who attained a legendary status in the history of neuroscience and psychology, occurred against a background of largely intact cognitive, intellectual, perceptual, social and sexual functions. E.L. neither exhibited emotional and behavioral changes, including disinhibition, jocularity, and decline in social and occupational functioning, nor changes in higher-level cognitive and other executive functions associated with Gage's accident and “classical damage” to ventromedial- and dorsolateral prefrontal regions. Multitasking assessments and TMS-targeted protocols, however, unmasked compensatory mechanisms through modulation of slow wave oscillations under single task conditions, to maintain motor performance and executive functioning, eluding clinical detection and underlying brain capability to restore ‘normal life’ function after unilateral frontal lesions.

We selected tests for the most common neuropsychological functions sensitive to damage in the frontal lobe^16^ (Table 1; Extended Table 1). It is notable that E.L.’s decision-making impairments were not detected by gold standard tests of cognitive assessment, such as, IGT, CBT, WCST, and the ROCFT.^16^ From a clinical perspective, we identified deficits in E.L. across working memory, including short-term memory, and using task switching tests, an executive function that involves the ability to unconsciously shift attention between dual-tasks. Similarly, we identified borderline performances on language (semantic [animals] and phonological [letter ‘p’]) tests (Table 1; Extended Table 1). Although cognitive impairments are not always evident on Divided Attention and on Working Memory tests, previous studies reported that patients with right hemisphere damage exhibited a worse performance,^23^ supporting the concept that the contribution of the right hemisphere is more pronounced than the left hemisphere for the processing of visuospatial short-term memory tasks. In agreement with this concept, E.L. showed working memory deficits, independent of a clear rising complexity level, and deficits on the spatial span, in agreement with previous reports on TBI patients.^24^ However, several confounding variables must be taken into account. Among them, are the side effect of some symptomatic treatments with OXC - an anticonvulsant, which can impair performance in cognitive functions-,^25^ intelligence,^26^ age, and degree of education. Accordingly, no differences in anti-saccade task and eye tracking performance, manifested by subjects with a variety of neurological and psychiatric conditions,^27^ were found on the OXC-treated CTRL and E.L. Thus, it remained unclear the extent to which a deficit in top-down inhibition in E.L. had functional significance.

Noting that dual-task interference effect may reflect the disruption of a central attention processor that uses limited resources to subordinate processing mechanisms for executing a task,^28^ we decided to perform imaging and electrophysiological assays in conjunction with memory-guided simple sensorimotor decision tasks. E.L.’s performance on simultaneous bimanual movements is illuminated by his cortical activation patterns during functional neuroimaging assays, debunking misinterpretations and shedding light on resource allocation during task performance. In particular, E.L.’s movements revealed that right hand finger tasks induced most activation in the contralateral PMA, PFC and Supplementary Motor area (SMA) and ipsilateral cerebellum, in terms of total/relative voxels relative to his SMA. Previous studies reported that the primary sensory-motor cortex (SM1) is more activated by finger than toe movements, although the PMA and PFC are more activated by toe movements.^29^ Therefore, different bold fMRI activation patterns are believed to be generated by movements of the arms or legs. On the other hand, we found that E.L.’s both unimanual finger movements (left hand) or bimanual movements induced similar patterns of activation in the bilateral frontal and parietal regions, known to be involved in working memory.^30^ Previous research indicated that transcallosal inhibition from the contralateral to ipsilateral hemisphere in sensorimotor area, in response to voluntary extension/flexion of single hands, could account for modulation of neural activity on normal subjects^31^ and that brain injured subjects presented abnormally increased transcallosal inhibition from the healthy hemisphere onto the injured side.^32^ Nonetheless, the fact that reduced connectivity can reroute signal through more intact commissural fibers, thereby maintaining enough contralateral connectivity,^33^ is consistent with previous results in context of how interhemispheric connectivity can be impaired without reducing cognitive or functional ability.^3^^,^^34^ fMRI and TMS findings and qEEG for E.L. unveiled that interhemispheric connectivity via the use of the anterior or the posterior commissures in lieu of the *C.C*., correlated with spontaneous brain activity or were measured in the presence of task demands, respectively. While some studies of typical interhemispheric coordination in neurologically intact subjects suggest entirely cortico-cortical connections, others implicate subcortical contributions.^35^ Another possibility is that subcortical structures such as the thalamus or the superior colliculus play a greater role than previously thought in defining the flow of information between cortical regions.^36^^,^^37^ This adjustment mechanism has been suggested in humans with agenesis of *C.C*.^34^ In support of this view, interhemispheric functional synchronization in the absence of direct *C.C.* connectivity has also been reported in species that lack *C.C*. or have a small *C.C.*, such as song birds and cetaceans.^38^

Although the same neuronal circuits may produce slow wave (SW) oscillation (1-4 Hz) in seizing brain and during sleep,^39^^,^^40^ the two conditions seems to rely on differential activation of networks. Previous reports showed that SW activity is associated with increase fMRI-BOLD activation responses in a spatially related brain area.^41^ However, while SW is thought to consolidate memory activity in normal subjects during deep sleep (NREM),^42^ SW in the seizing brain is considered an epiphenomenon leading to dysfunction, including impairment of memory. The logic here has been that delta oscillation in lesioned and contralateral areas of E.L.’s brain may be not merely a marker of network dysfunction,^43^^,^^44^ but, instead, an expression of signalling rearrangement enabling performance.^12^ While E.L. presented a similar modulation of SW for passive/active foot movements, as predicted from previous work on Bold fMRI,^45^ his active right finger movements, by engaging his left hemisphere, led to increased SW in his right frontal cortex as shown by EEG recordings. Subsequent, single-handed left finger movements, engaging both hemispheres, decreased SW on his injured right hemisphere. That is, while a decrease of frequency of irregular SW in the right damaged hemisphere, assumed as a non-epileptiform activity, facilitated E.L. to accomplish a number of cognitive and motor tasks, persistence/increase of delta waves became an indicator of impairment/dysfunction. Different patterns of anatomical activation induced by E.L's fingers or foot movements explain lack of modulation of frequency of SW by the latter. On the other side, although a similar pattern of brain representation is elicited by active or passive movements for upper limbs, during active movements only, activations of the basal ganglia and the cingulate gyrus were found.^46^

TMS is increasingly used to create ‘virtual lesions’, with temporospatial resolution, in selected brain regions.^47^ Thus, temporary disturbance of task-related neuronal activity mediated by a specific region should yield a decline in executive performance and behavior. We tested this prediction by applying rTMS pulses over the left PMC (motor), dlPFC (working memory) and vmPFC (behaviour)^48^ and showed that it is possible to reliably trigger SWs in awake injured brain that resemble in all aspects spontaneously TMS-induced slow oscillations during NREM sleep (i.e., state-dependent).^49^ Spatially, the TMS-treatment resulted in a substantial increase in SW oscillations that spread antero-to-posterior like a ‘water ripple wave’ across E.L.’s right hemisphere and laterally to his contralateral frontal lobe during wakefulness. In contrast to E.L. awake brain, however, the hot spot for TMS-triggered oscillations on CTRL sleep NREM brain corresponded closely to a hot spot for the origin of spontaneous SWs and their sensorimotor regions constituted a preferential site for triggering such SWs.^50^ In contrast to E.L., TMS-triggered frontal SW oscillations in sleeping heathy brain are state-specific and displayed a sudden disappearance after transition from NREM to wakefulness.^49^ Thus, lack of TMS-triggered SWs on our awake CTRL was not unexpected. By demonstrating these findings in awake injured brain, however, our study suggests that in contrast to the ‘sleeping mode’,^13^ E.L.’s injured brain, active and reactive, does not lose its ability of entering integrated and differentiated states because of prompt modulation of frequency and amplitude of SW oscillations by the left contralateral hemisphere. Results from the present study support that such features are critical for performance of E.L. executive functions. It is important to note that, regardless of stimulation site, TMS pulses applied to E.L scalp area, were equally effective overlying PMC, dlPFC and vmPFC. Thus, in contrast to the sensorimotor cortex (precentral and postcentral gyri areas) on ‘sleeping brain’,^49^ where activation of corticoreticulothalamocortical circuits by TMS primarily support triggering of SWs, evidence from our study support that, in injured E.L. awake brain, contralateral cortical reactivity is required to attenuate the frequency of irregular SW activity. Following the anticipated modulation criteria of contralateral SWs oscillation, the second criterion of successful compensation required disturbance of executive functioning and behaviour on selected regions, and a consequent decline in specific performance upon TMS-treatment. Temporary maintenance and processing of information, involving executive processes that manipulate the contents and retention of working memory, was explored assessing the ROCF task. Several studies give evidence for a role of the left dlPFC in working memory, but, no apparent deficits manifested by E.L. on any outcome measures (copy, IR and DR trials of ROCF test) were suggested prior to TMS-pulses as well as no effects were observed following unilateral stimulation of CTRL on any outcome measures.^51^ We tested this prediction by probing the reactivity and executive functioning in E.L and CTRL brain after applying TMS pulses in left dlPFC at intensities commonly used in clinical practice.^52^ As predicted, consolidation of the Rey-Osterrieth Complex Figure was disrupted at both 3 min (IR) and 30 min (DR) by stimulation selectively over E.L. dlPFC, without disruption of his motor skills or other aspects of motor function. Such strategy failed to find a similar disruption of executive functioning in the CTRL, suggesting that bilateral ‘lesions’ are required to impair short-memory consolidation and retrieval. In a third approach, an increase of SWs over the right hemisphere by means of TMS protocols applied to left vmPFC, used to interfere with motivation (i.e., apathy and asociality), disrupted E.L. willingness to engage in social interaction, similar to disorders secondary to bilateral frontal damage,^1^ and produced indifference regarding interpersonal relationship. In contrast to E.L., the CTRL group did not manifest change in behaviour after TMS-treatment. Return to normal behaviour paralleled progressive reduction of SW activity over E.L. right hemisphere, within 60 min of TMS-stimuli.

Few cases in the history of neurosciences have been reported as frequently as the TBI case of Phineas Gage, which still lives as a part of neuroeducation. While great attention is given to executive dysfunction, one can merely speculate how Gage's brain injury actually affected his daily routine and feasibility of functional compensation. The current E.L. case, whose brain injury mirrored that of Gage's, sheds light on SW oscillatory dynamics following TBI, unveils compensatory mechanisms by which PFC may accommodate executive functional performance, which frequently elude clinical diagnosis, and provides an attractive target for therapeutic interventions on cortical circuits.

## Contributors

R.C.M.S.F., F.A.V., I.S.D., R.M.B. and C.M.P. performed neurosurgical procedures. C.T.F.T., C.C., J.T., K.P. and E.G. selected neuropsychological batteries and performed testing and interpretations. L.C.H.C. Jr. performed diagnostic MRI. G.F.G. and F.M. applied BIQS testing. G.L.W. provided advice on statistical methods. M.F. and P.H.F. performed EOG testing. P.H.F., R.B., G.F.G., S.F.A.R., C.M.P. and R.R. developed the figures, table and movies; analysed data and revised the manuscript. W.S.P., C.Y.H., P.C.R. and P.H.F. conducted the T.M.S. assays. P.H.F., C.M.P., J.V., T.M.M.L., M.M.S., F.L., H.J.F. and R.R. performed neurological and neurophysiological assessments. J.R.L.S. developed the 3D brain modelling. D.H.P. performed diagnostics assays. R.L., M.V.L.B., P.H.F. and R.R. - conceived the study, structured its design, interpreted and integrated data and organized the general discussion. M.V.L.B. and R.R. - drafted and edited the main manuscript and proved the final version.

## Declaration of interests

All authors declare that they have no conflicts of interest.

## Appendix Supplementary materials

EXTENDED DATA Extended FIG. 1 (a) 3D CT reconstruction of the iron bar trajectory through E.L.’s skull. (b,c) View of the large craniotomy allowing for a pterional-type approach to the iron bar within the intracranial cavity, exposing brain laceration and the perforated bone flap. (b) Note the path drilled by the iron bar in bone as it passed through the skull. (d,e) Lateral section MRI images depicting the path of the iron bar, pointing the entry- and exit-points with white arrow and arrowhead , respectively. (f) Coronal section MRI scan depicting direct damage to the right side of the nose and right frontal sinus secondary to the passage of the iron bar. (g,h) Lateral and axial images from a follow-up MRI scan performed 18 months later help propose a right frontal lobe disconnection (white arrows). R (right hemisphere), L (left hemisphere). Image, image 1

Extended FIG. 2. MRI follow-up of patient E.L. T2 weighted sequences suggesting the penetrating lesion extension. a, c – Scan sequences obtained 12 days after the transfixing traumatic brain insult, with overt perilesional edema (white arrow), without midline deviation. b, d – 18 months follow-up, with no visible edema, atrophy, or neurodegenerative signs. Axial (a, b) and coronal (c, d) sections, respectively. Image, image 2

Extended FIG. 3. E.L.: Failure of compensatory mechanisms during bimanual challenge. Coordination testing showed symmetrical movements without dysmetria, or incoordination, on finger to nose testing, normal forearm (a) and finger rolling tests (b), in which the subject rotates just the index fingers using rapidly alternating movement, and on sequential active finger-to-thumb tapping with the right hand alone (c) or with the left hand alone (d). However, there was incoordination of sequential finger-to-thumb tapping movements in the left hand during bimanual movements (e) low-magnification and (f) higher magnification. In the beginning, E.L. expressed surprise at the outcome to see that his left-hand fingers could end up paralyzed for a few sec upon the execution of bimanual movements. Image, image 3

Extended FIG. 4. 3D brain reconstruction from T1 MRI scan sequences helped unveil E.L.’s right frontal lobe dysfunction. Cortical surfaces reconstruction with sulcal identification output by FreeSurfer (inset) are illustrated (a,b). Life-size 3D view metrics of E.L brain morphology and the path that the transfixing iron bar followed through his right hemisphere (a,b in red colour), printed in polyamide (c), highlighted compromised regions and presented a detailed picture of the extent of the damage. R (right hemisphere), L (left hemisphere). Calibration bar (c): 2 cm. Image, image 4

Extended FIG. 5. Visual representation of major white matter fiber tracts tracked in E.L. Diffusion tensor tractography quantification of Uncinate Fasciculus (yellow), Cingulum bundle (blue) and Superior Longitudinal Fasciculus (SLF) (green), compromised in E.L. accident, and Corpus Callosum (*C.C.*) (red), are illustrated in lateral and axial scans (a-f). (c,d,e). Axial and lateral views of a tractography suggested a difference in the volume of the association fiber tracts that connect temporo-parietal cortical regions with right frontal lobe (b,c,f – white arrows), but no difference in the volume of the C.C. association fiber tracts that connect the frontal lobes (c,e,f – red). Image, image 5

Extended FIG. 6. E.L.’s performance in Iowa Gambling Task (IGT), used to assess risk-based decision-making and impulsivity after TBI, is sensitive to behavioural deficits in subjects with PFC damage. The test consists of a card game where the risks and rewards vary by the decks chosen. (a) Percentage of cards selected from different decks. (b) Two of these decks (decks A [highest risk deck] and B) have higher short-term payoffs (‘high risk’) than the other two (decks C and D) (advantageous ‘safe’ decks), but over time (10 trials of the IGT) the decks with high immediate payoffs are disadvantageous, resulting in a long-term loss (net loss). (c,d) E.L.’s score based in safe vs. risky decks and numbers of cards selected (safe vs risky decks) over the course of the test. (e) reaction times (in sec) for each trial (total 100 trials) of the test (deck choice represented by colour patterns depicted in ‘a’). Total net IGT scores (100 trials per session) is analysed using methods of scoring risky decisions (Methods). Complex emotion based-learning remained intact in E.L. Image, image 6

Image, image 7

Extended FIG. 7a,b. Assessment of sexuality following traumatic brain injury (TBI). The Brain Injury Questionnaire on Sexuality (BIQS), which is designed to account for temporal changes in sexual function, was applied in parallel to E.L. and to L., his wife (a, b), 31 months following E.L.’s TBI. Image, image 8

Extended FIG 8. The antisaccade task and the voluntary control of eye movement. Saccadic and antisaccadic eye movement tasks depend on the frontal/prefrontal cortex and related structures. (a) Schematic diagram of EOG test. A symbolic cue, such as a colour dot, instructs the subject to make a brief, rapid eye movement (a saccade) towards the stimulus (prosaccade) (green) or in the opposite direction (antisaccade) (red). (b) The schematics of the overlap (top layer) and gap (middle layer) tasks performed are displayed in msec. (Bottom) Reaction time latencies of eye movements during prosaccade and antisaccade commands. (c,d) Performance of a CTRL and E.L. during a gap task. (c) Eye position traces during prosaccades (left). (middle, right) Distribution of reaction times for each participant (histogram) – single test. (d) Antisaccade tasks*.* Correct responses are displayed in blue, corrected errors in red and anticipations or uncorrected errors in black – single test. (e,f) Overlap task % and gap task % distribution of EOG responses for the CTRL and E.L. Differing effects of prosaccades (Pro) and antisaccades (Anti) are illustrated by histograms correspondent to right (top) or left (bottom) stimuli. Image, image 9

Extended FIG. 9. Impaired antisaccades in oxcarbazepine-treated subjects. The comparison between the outcome of eye-tracking performance in oxcarbazepine (OXC)-treated E.L. and in OXC-treated/untreated CTRL (*n* = 5). (a) The first row depicts latencies in saccade, prosaccade and antisaccade paradigms, presented randomly to the right or the left side. (b) The second row displays the performance (correct rate responses) of the OXC-treated subjects and CTRL. * - *P* ≤ 0.05. (b) Mean ±SEM values of prosaccade and antisaccade and latency of all groups. Of note, there were no differences in symmetry (right vs left, or up vs down) of prosaccadic-antisaccadic latency, velocity, or gain between E.L. and the OXC-treated CTRL. (c) E.L.’s therapeutic plasmatic levels of oxcarbazepine. Image, image 10

Extended TABLE 1. Neuropsychological battery performed in E.L. Abbreviations: MMSE: Mini Mental State Examination; BDI: Beck's Depression Inventory; WCST: Wisconsin Card Sorting Test (Modified Card Sorting Test. Nelson, 1976); PM 38: Standard Progressive Matrices 1938; RCFT: Rey – Osterreith Complex Figure Test; TMT: Trail Making Test; Stroop-W: word reading Stroop Task; Stroop-C: colour naming Stroop Task; Stroop CW: Colour-word interference Stroop Task; DMS-48: Delayed Matching to sample (Barbeau, 2004); CVLT: California Verbal Learning Test; BNT: Boston Naming test; Facial Recognition Test (Benton, 1983) Image, application 11

SUPPLEMENTARY MOVIES Movie #1. Supplementary movie S1. E.L.’s challenge: finger-to-thumb movement performed simultaneously by both hands. Image, video 12

Movie #2. Supplementary movie S2. 3D rendering of a T1-weighted MRI anatomical scan of E.L.’s brain. Image, video 13

Movie #3. Supplementary movie S3. 3D rendering of a T1-weighted MRI anatomical scan of E.L.’s Corpus callosum. Image, video 14

Supplementary Clinical Case History. Image, application 12

Supplementary Methods. Image, application 13
