# Supplementary figures and images for: E.L., a modern-day Phineas Gage: Revisiting frontal lobe injury
Source: Lancet Reg Health Am. 2022 Aug 11;14:100340. doi: 10.1016/j.lana.2022.100340 (PMC9903712; doi:10.1016/j.lana.2022.100340)

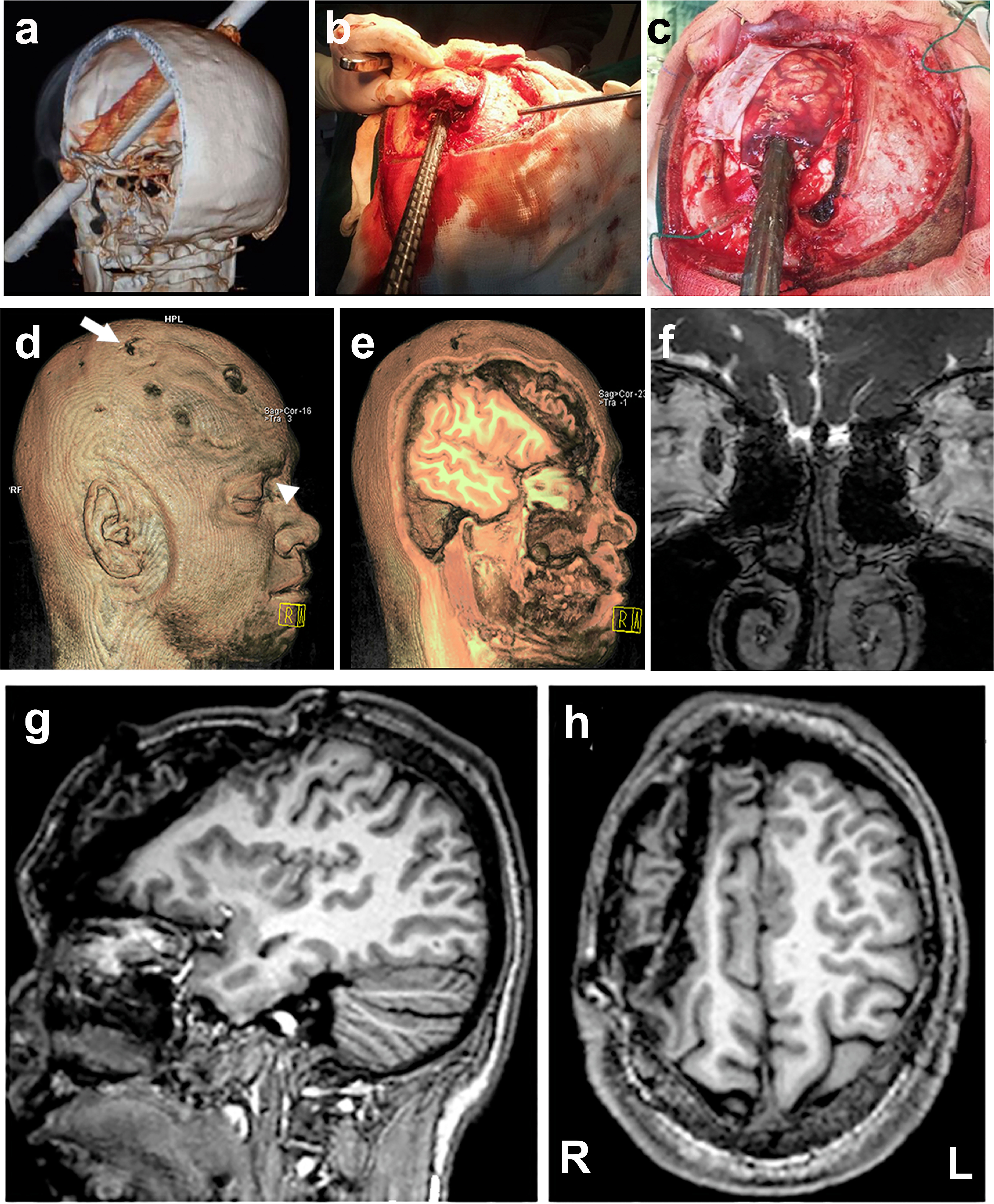

Supplement: Supplementary file 1 — EXTENDED DATA Extended FIG. 1 (a) 3D CT reconstruction of the iron bar trajectory through E.L.’s skull. (b,c) View of the large craniotomy allowing for a pterional-type approach to the iron bar within the intracranial cavity, exposing brain laceration and the perforated bone flap. (b) Note the path drilled by the iron bar in bone as it passed through the skull. (d,e) Lateral section MRI images depicting the path of the iron bar, pointing the entry- and exit-points with white arrow and arrowhead , respectively. (f) Coronal section MRI scan depicting direct damage to the right side of the nose and right frontal sinus secondary to the passage of the iron bar. (g,h) Lateral and axial images from a follow-up MRI scan performed 18 months later help propose a right frontal lobe disconnection (white arrows). R (right hemisphere), L (left hemisphere). [file mmc1.jpg]

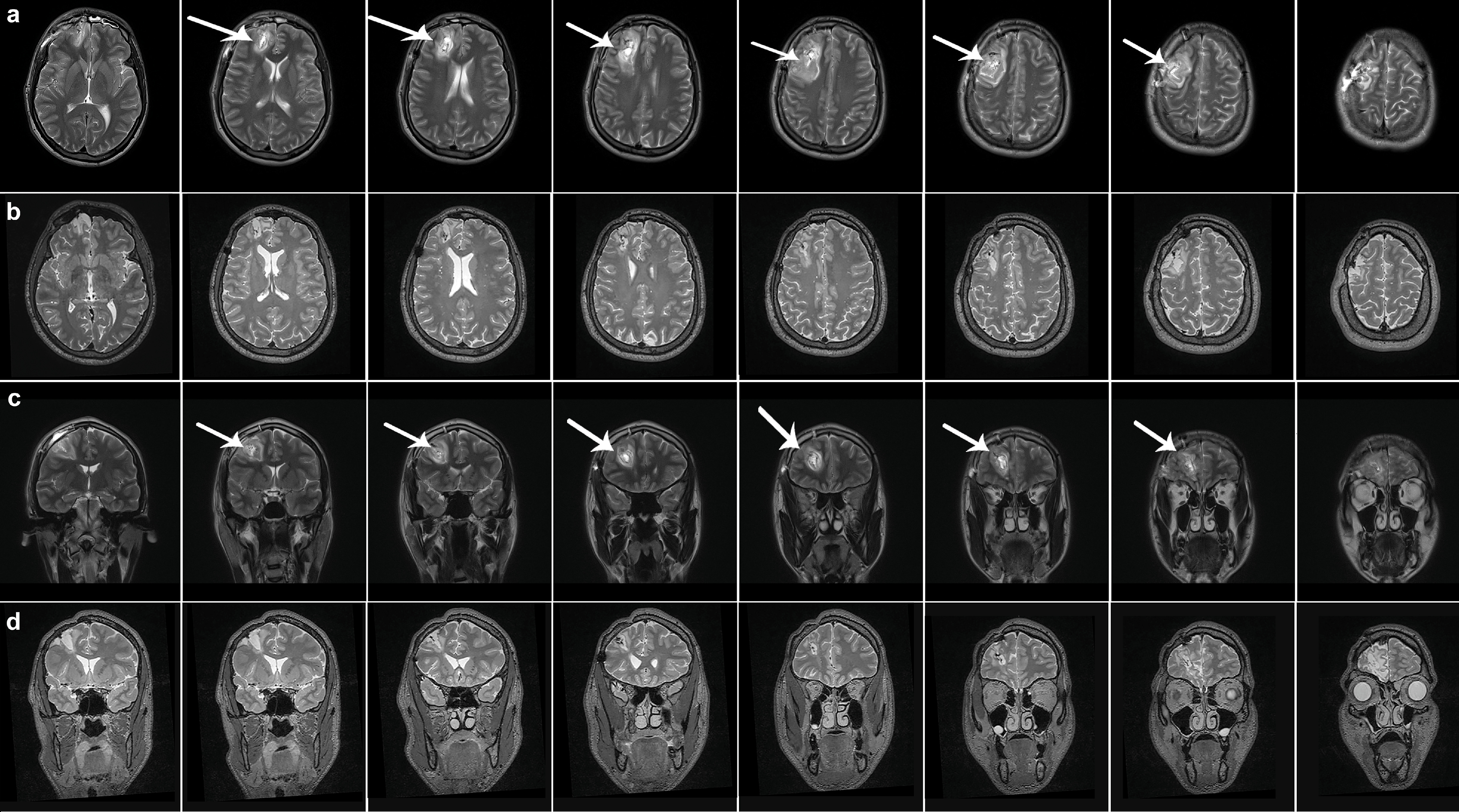

Supplement: Supplementary file 2 — Extended FIG. 2. MRI follow-up of patient E.L. T2 weighted sequences suggesting the penetrating lesion extension. a, c – Scan sequences obtained 12 days after the transfixing traumatic brain insult, with overt perilesional edema (white arrow), without midline deviation. b, d – 18 months follow-up, with no visible edema, atrophy, or neurodegenerative signs. Axial (a, b) and coronal (c, d) sections, respectively. [file mmc2.jpg]

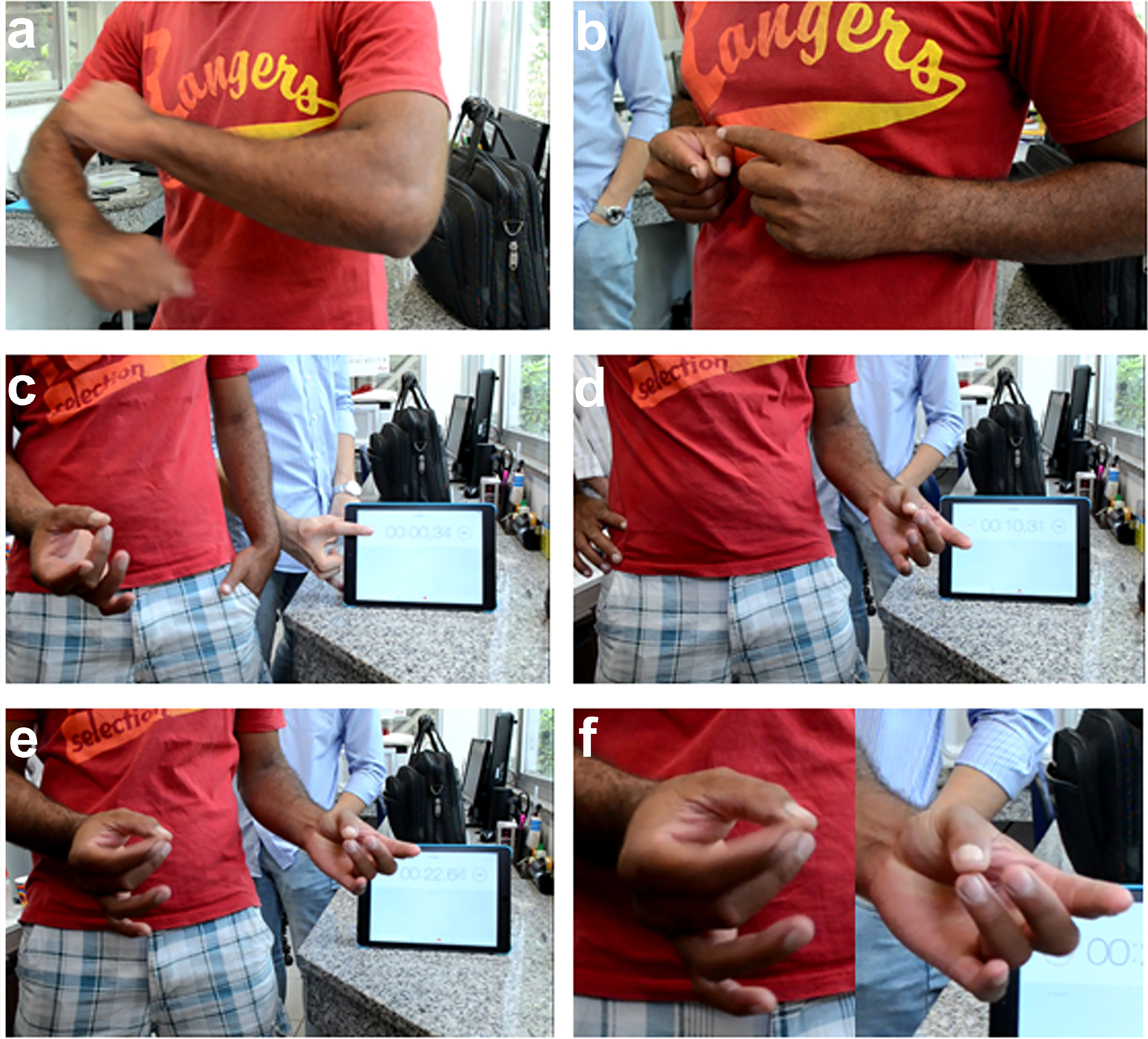

Supplement: Supplementary file 3 — Extended FIG. 3. E.L.: Failure of compensatory mechanisms during bimanual challenge. Coordination testing showed symmetrical movements without dysmetria, or incoordination, on finger to nose testing, normal forearm (a) and finger rolling tests (b), in which the subject rotates just the index fingers using rapidly alternating movement, and on sequential active finger-to-thumb tapping with the right hand alone (c) or with the left hand alone (d). However, there was incoordination of sequential finger-to-thumb tapping movements in the left hand during bimanual movements (e) low-magnification and (f) higher magnification. In the beginning, E.L. expressed surprise at the outcome to see that his left-hand fingers could end up paralyzed for a few sec upon the execution of bimanual movements. [file mmc3.jpg]

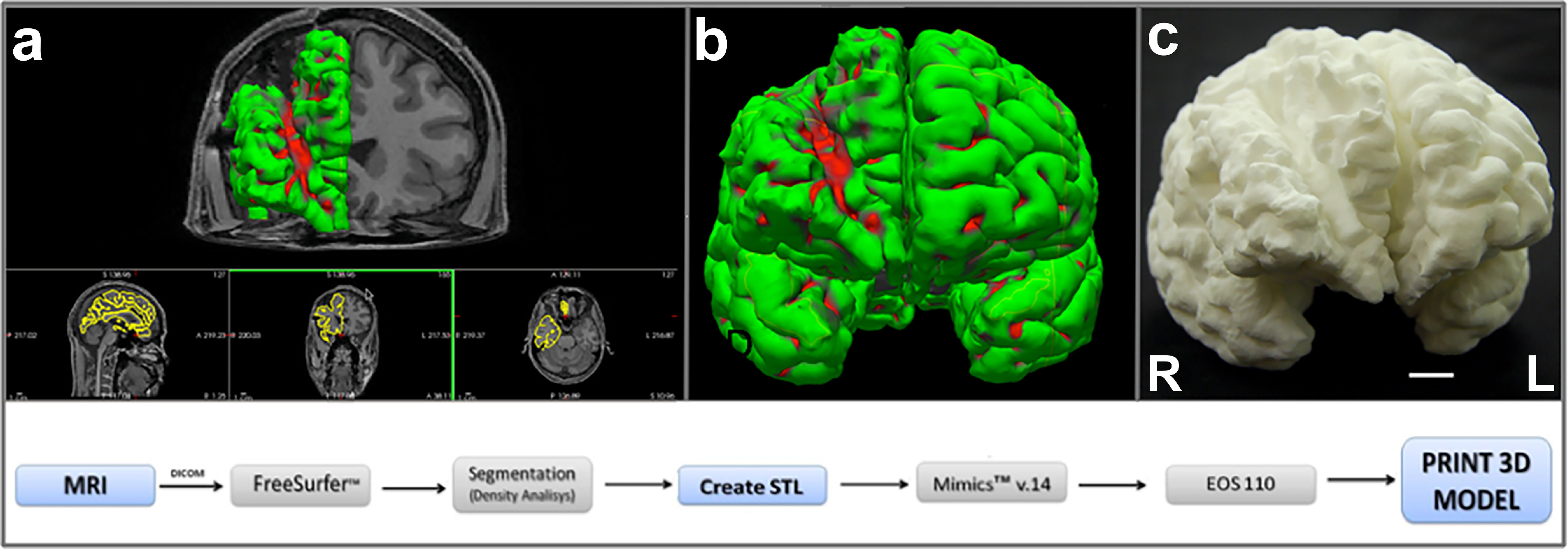

Supplement: Supplementary file 4 — Extended FIG. 4. 3D brain reconstruction from T1 MRI scan sequences helped unveil E.L.’s right frontal lobe dysfunction. Cortical surfaces reconstruction with sulcal identification output by FreeSurfer (inset) are illustrated (a,b). Life-size 3D view metrics of E.L brain morphology and the path that the transfixing iron bar followed through his right hemisphere (a,b in red colour), printed in polyamide (c), highlighted compromised regions and presented a detailed picture of the extent of the damage. R (right hemisphere), L (left hemisphere). Calibration bar (c): 2 cm. [file mmc4.jpg]

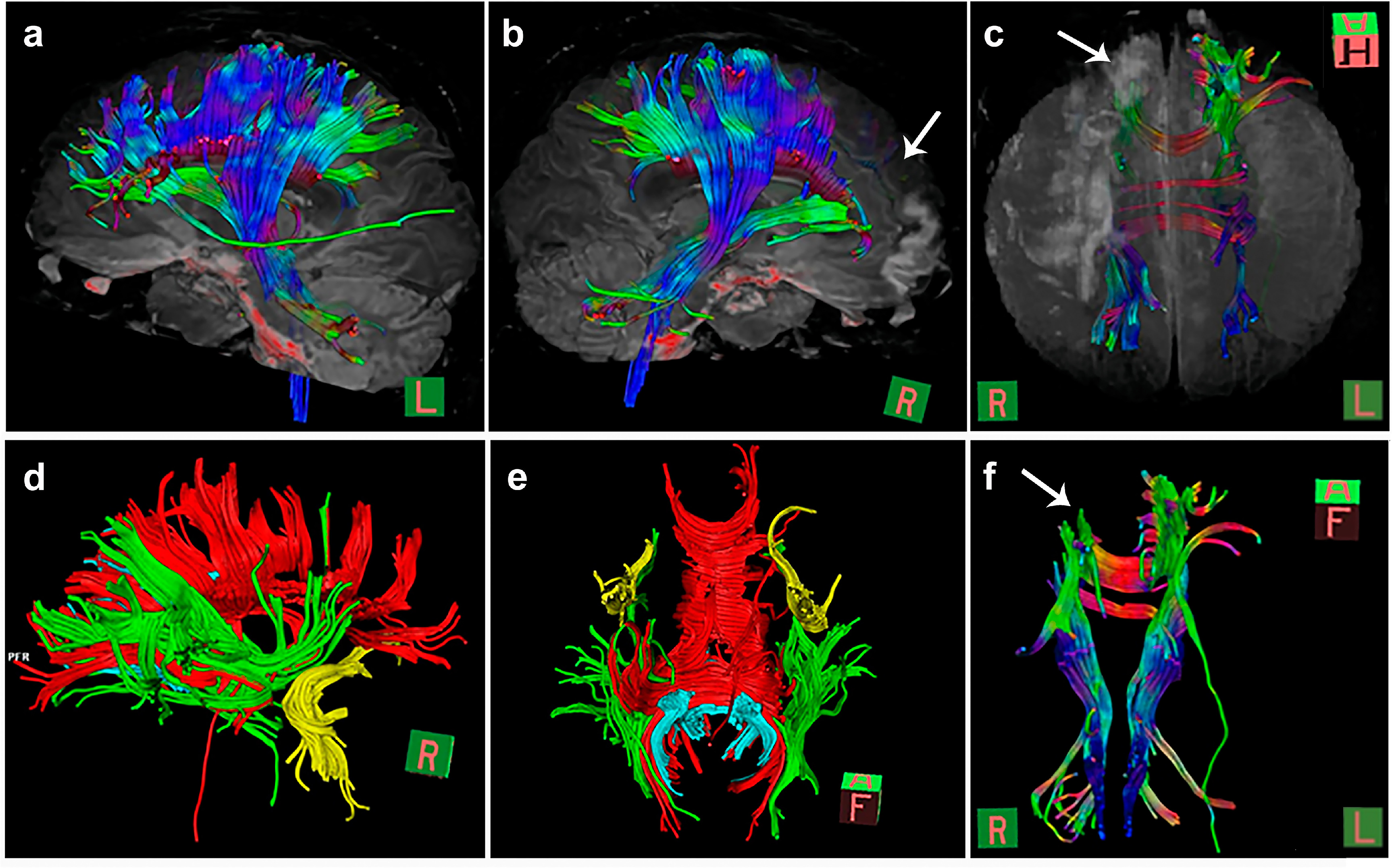

Supplement: Supplementary file 5 — Extended FIG. 5. Visual representation of major white matter fiber tracts tracked in E.L. Diffusion tensor tractography quantification of Uncinate Fasciculus (yellow), Cingulum bundle (blue) and Superior Longitudinal Fasciculus (SLF) (green), compromised in E.L. accident, and Corpus Callosum (C.C.) (red), are illustrated in lateral and axial scans (a-f). (c,d,e). Axial and lateral views of a tractography suggested a difference in the volume of the association fiber tracts that connect temporo-parietal cortical regions with right frontal lobe (b,c,f – white arrows), but no difference in the volume of the C.C. association fiber tracts that connect the frontal lobes (c,e,f – red). [file mmc5.jpg]

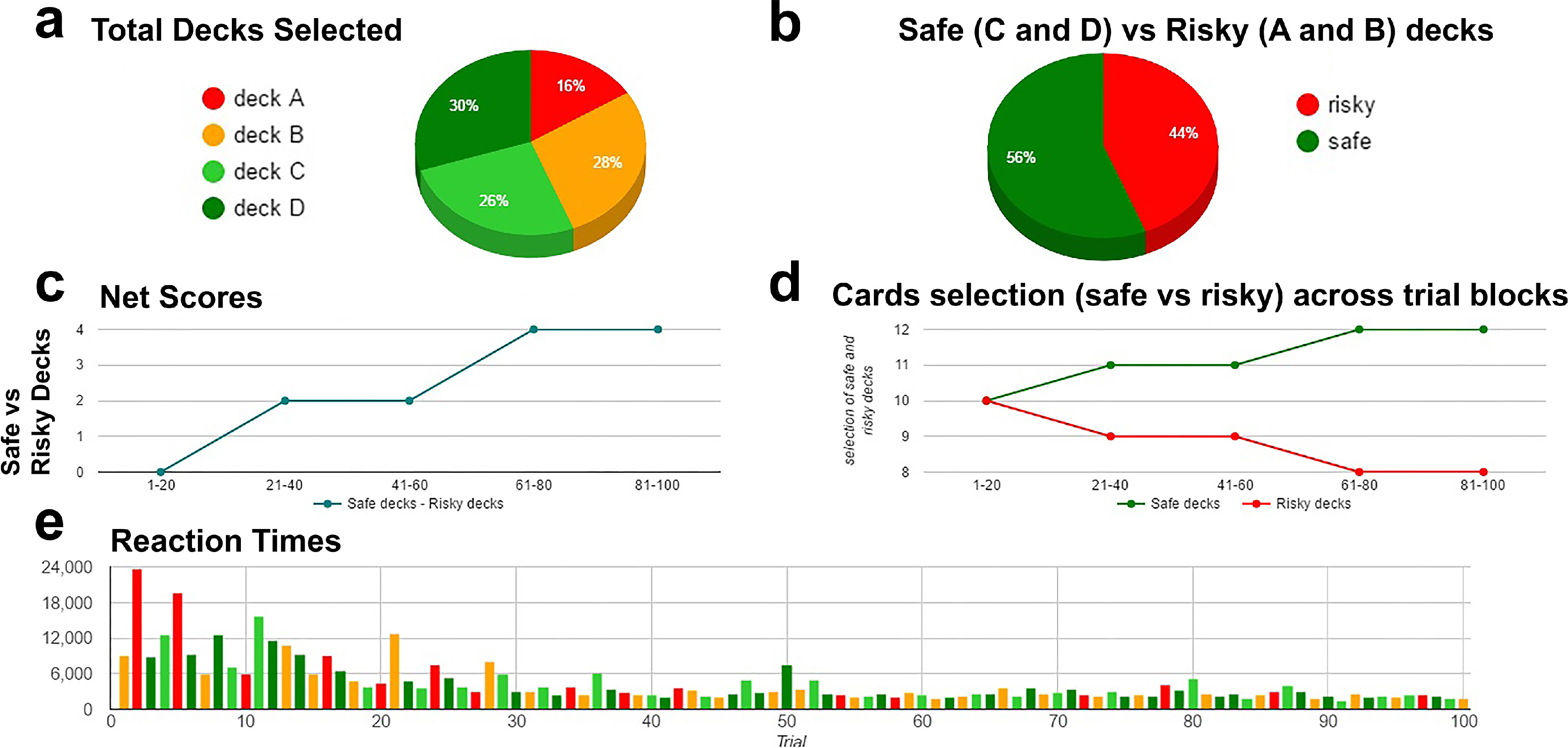

Supplement: Supplementary file 6 — Extended FIG. 6. E.L.’s performance in Iowa Gambling Task (IGT), used to assess risk-based decision-making and impulsivity after TBI, is sensitive to behavioural deficits in subjects with PFC damage. The test consists of a card game where the risks and rewards vary by the decks chosen. (a) Percentage of cards selected from different decks. (b) Two of these decks (decks A [highest risk deck] and B) have higher short-term payoffs (‘high risk’) than the other two (decks C and D) (advantageous ‘safe’ decks), but over time (10 trials of the IGT) the decks with high immediate payoffs are disadvantageous, resulting in a long-term loss (net loss). (c,d) E.L.’s score based in safe vs. risky decks and numbers of cards selected (safe vs risky decks) over the course of the test. (e) reaction times (in sec) for each trial (total 100 trials) of the test (deck choice represented by colour patterns depicted in ‘a’). Total net IGT scores (100 trials per session) is analysed using methods of scoring risky decisions (Methods). Complex emotion based-learning remained intact in E.L. [file mmc6.jpg]

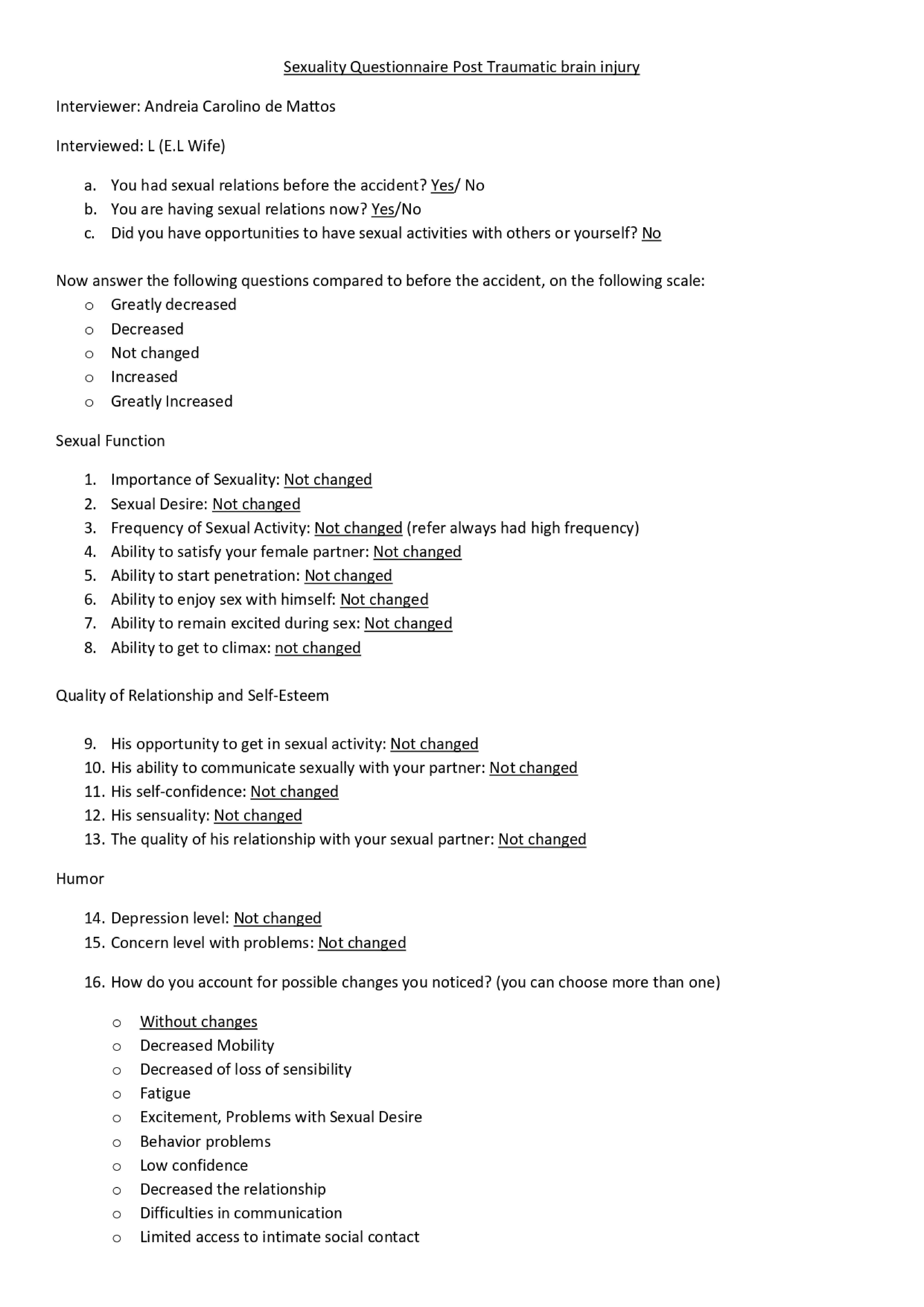

Supplement: Supplementary file 7 [file mmc7.jpg]

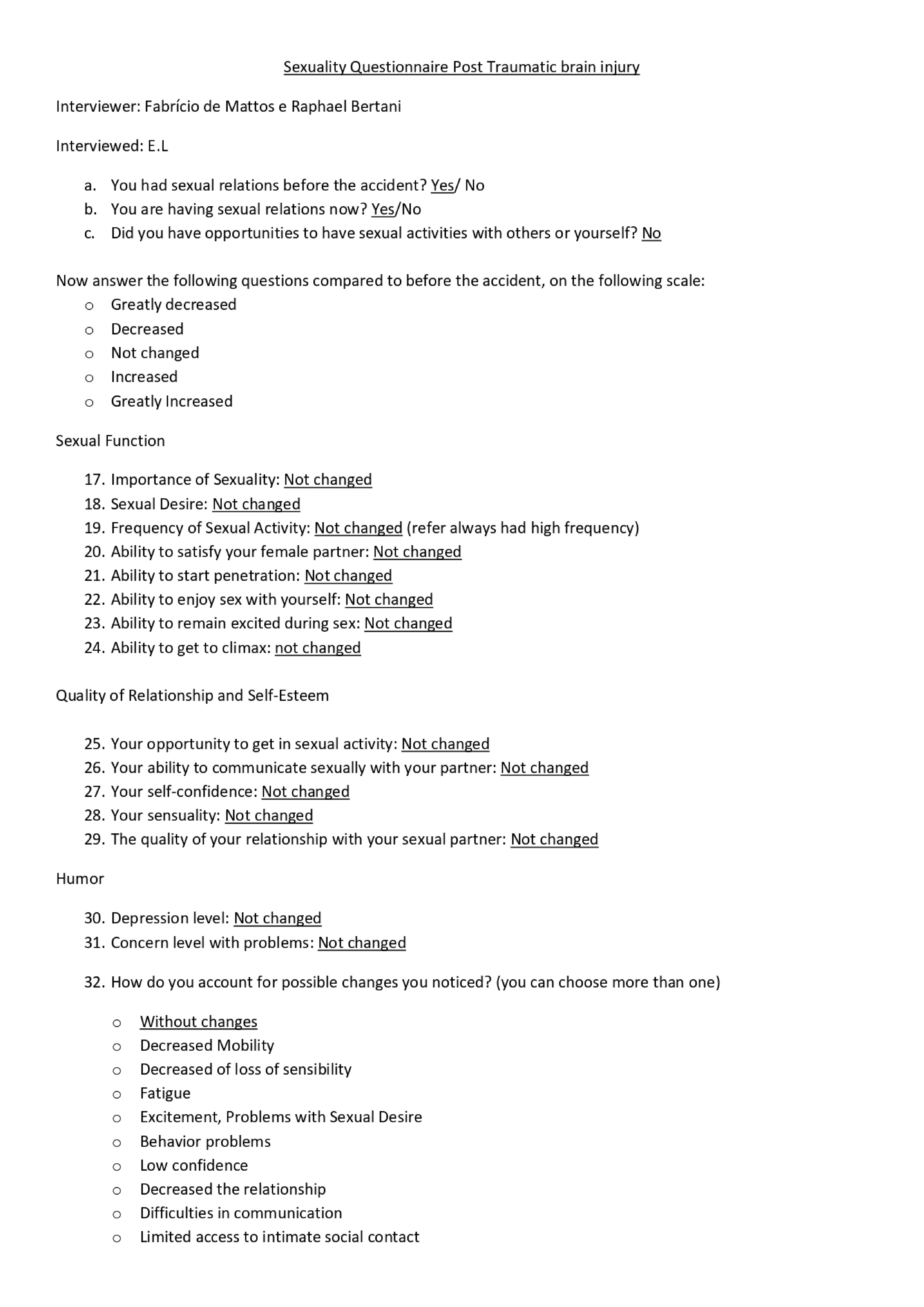

Supplement: Supplementary file 8 — Extended FIG. 7a,b. Assessment of sexuality following traumatic brain injury (TBI). The Brain Injury Questionnaire on Sexuality (BIQS), which is designed to account for temporal changes in sexual function, was applied in parallel to E.L. and to L., his wife (a, b), 31 months following E.L.’s TBI. [file mmc8.jpg]

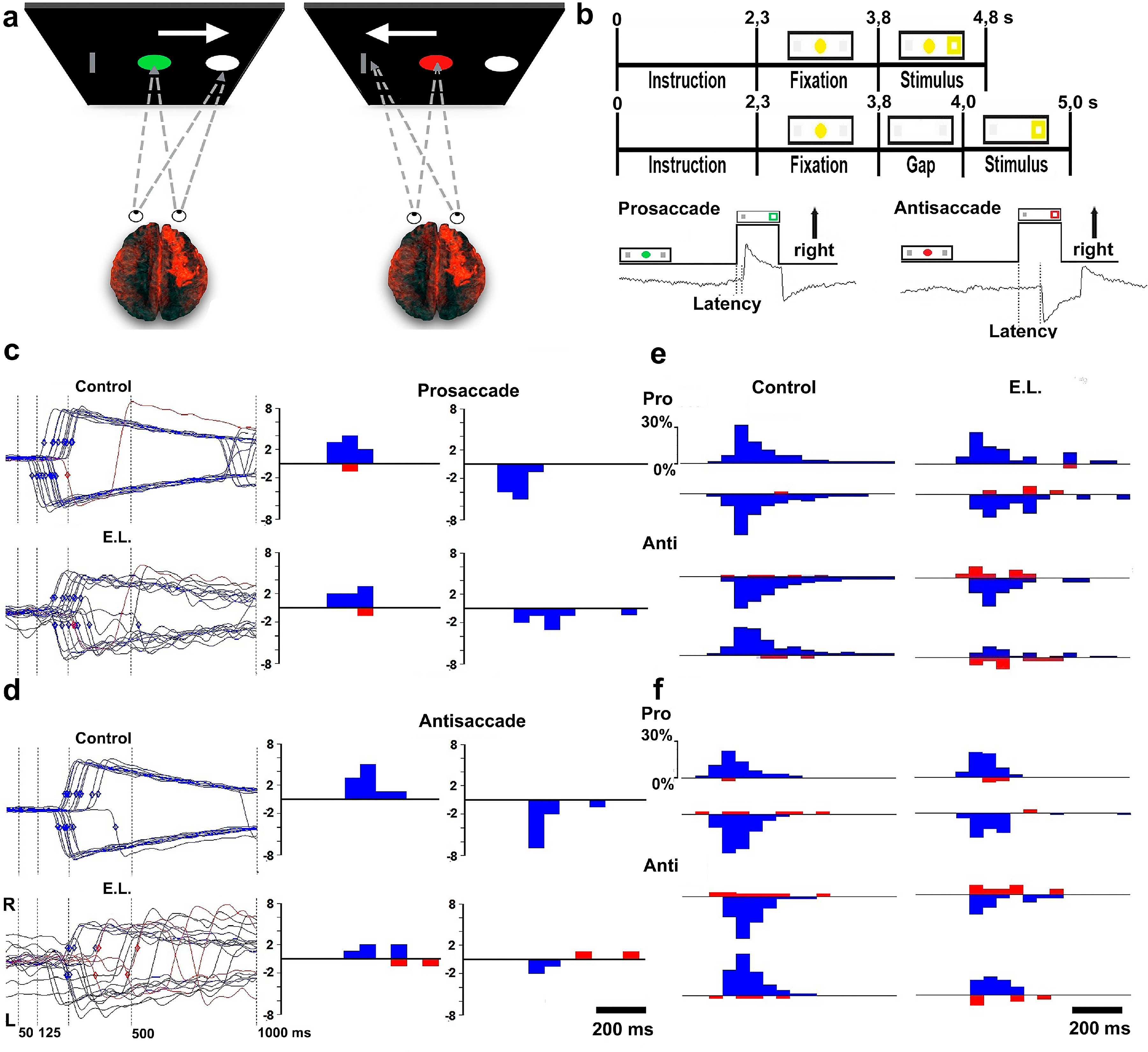

Supplement: Supplementary file 9 — Extended FIG 8. The antisaccade task and the voluntary control of eye movement. Saccadic and antisaccadic eye movement tasks depend on the frontal/prefrontal cortex and related structures. (a) Schematic diagram of EOG test. A symbolic cue, such as a colour dot, instructs the subject to make a brief, rapid eye movement (a saccade) towards the stimulus (prosaccade) (green) or in the opposite direction (antisaccade) (red). (b) The schematics of the overlap (top layer) and gap (middle layer) tasks performed are displayed in msec. (Bottom) Reaction time latencies of eye movements during prosaccade and antisaccade commands. (c,d) Performance of a CTRL and E.L. during a gap task. (c) Eye position traces during prosaccades (left). (middle, right) Distribution of reaction times for each participant (histogram) – single test. (d) Antisaccade tasks. Correct responses are displayed in blue, corrected errors in red and anticipations or uncorrected errors in black – single test. (e,f) Overlap task % and gap task % distribution of EOG responses for the CTRL and E.L. Differing effects of prosaccades (Pro) and antisaccades (Anti) are illustrated by histograms correspondent to right (top) or left (bottom) stimuli. [file mmc9.jpg]

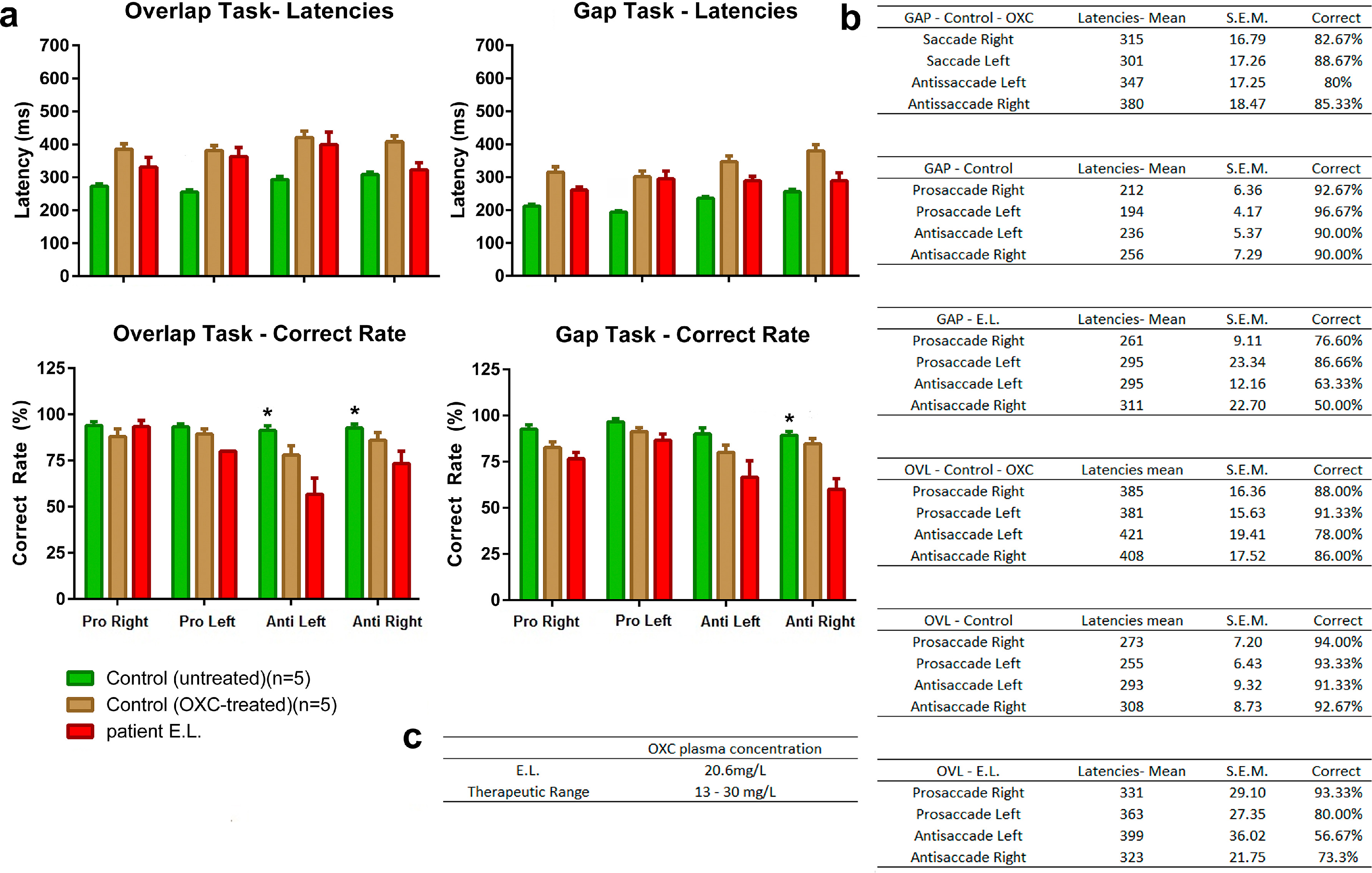

Supplement: Supplementary file 10 — Extended FIG. 9. Impaired antisaccades in oxcarbazepine-treated subjects. The comparison between the outcome of eye-tracking performance in oxcarbazepine (OXC)-treated E.L. and in OXC-treated/untreated CTRL (n = 5). (a) The first row depicts latencies in saccade, prosaccade and antisaccade paradigms, presented randomly to the right or the left side. (b) The second row displays the performance (correct rate responses) of the OXC-treated subjects and CTRL. * - P ≤ 0.05. (b) Mean ±SEM values of prosaccade and antisaccade and latency of all groups. Of note, there were no differences in symmetry (right vs left, or up vs down) of prosaccadic-antisaccadic latency, velocity, or gain between E.L. and the OXC-treated CTRL. (c) E.L.’s therapeutic plasmatic levels of oxcarbazepine. [file mmc10.jpg]
