# Supplementary material for: E.L., a modern-day Phineas Gage: Revisiting frontal lobe injury
Source: Lancet Reg Health Am. 2022 Aug 11;14:100340. doi: 10.1016/j.lana.2022.100340 (PMC9903712; doi:10.1016/j.lana.2022.100340)
Supplement: Supplementary file 15 — Supplementary Clinical Case History. [file mmc15.docx]

**Supplementary Clinical Case History**

***Preamble***

While attempts to characterize Gage’s dysfunctions have been raising more questions than answers, little consideration has been given to the course of the recovery of his personality after the accident^1^. Lack of animal models for study of PFC injury and comparison is merely part of the conundrum.

***History and Physical Examination: ‘Replicating Gage’***

The round steel bar impaled E.L.’s skull obliquely on the right side, with an estimated kinetic energy of 588J, punching a perfect hole into the skull and transfixing the head (**Fig. 1a,b, Extended Data Fig. 1a-e**). The impact of the bar stunned E.L. at 9:30 am, but he did not lose consciousness at that time, or at any moment afterwards. E.L was conscious and talking as firefighters sawed through the bar. The penetrating portion of the bar was stabilized by the emergency firefighters to minimize movement and brain damage during E.L.’s transportation to Hospital Municipal Miguel Couto (HMMC), a Level I Trauma Center, Rio de Janeiro. He was transported to the hospital with half a meter of the rod left in his head. E.L. was admitted to the ER at Hospital Municipal Miguel Couto (HMMC) at 10:17 am; at admittance his Glasgow Coma Score (GCS) was ‘mild’ (i.e., GCS of 15), he received computerized tomography (CT) scans of the brain, had a blood specimen collected for biochemistry and serology and reached the operating room (O.R.) at 11 am, for the removal of the iron rod and exploration of the facial wounds by neurosurgical and oral & maxillofacial surgery teams.

E.L. was placed supine on the operating table; his head was placed in Mayfield pins with head turned leftward 70°. While E.L.´s spiral ribbed steel bar fell and transfixed his skull (**Extended Data Fig. 1b**), which accounted for the development of a well-defined circumscribed right frontal lesion, assessed by imaging. Gage´s projectile, without a sharp edge, is believed to have penetrated by way of kinetic energy deposition, crushing tissue along the way. Low cerebral bleeding during E.L. transportation to the hospital was credited to hemostasis supported by the physical contact with the steel bar, which was removed frontward at the OR to prevent retrograde infection with pathogens from the damaged right olfactory region. E.L.´s satisfactory evolution in terms of post-traumatic insult was credited, in part, to brain hypothermia during the procedure for removal of the steel bar and subsequently and the prompt control of infection with antibiotics.

In the ICU, E.L. was placed on a double antibiotic broad-spectrum antibiotics regimen for 10 days for prophylaxis against microorganisms. On day 11, E.L. was discharged from the ICU to a medical-surgical ward. Throughout this period of time, he did not experience any weakness, change in sensation, or loss of vision (GCS of 15 postoperative). Throughout his hospitalization, he did not note any change in his strength or sensation and underwent an MRI brain scan, revealing injury of the right frontal lobe, a marked perilesional edema without midline shift (**Extended Data Fig. 2a,c**).

E.L. returned to work on a construction site 3 months after the accident and participated in a full range of normal activities, including operating heavy equipment and working at elevated locations. He followed usual safety precautions, and noted no change in his balance or strength through this time. He also continued functioning normally for approximately 9 months after his return to work, until he experienced a tonic-clonic seizure.

**Neurological Examination**

Seizure semiology: E.L. developed PTE 9 months after the accident. Consciousness was impaired. He experienced motor seizures 11 times (72% within the first 23 months of treatment), all lasting less than 1 min, and starting out with shaking of the left arm, and then spreading to involve his whole left side, before involving both sides of his body. All episodes were due to medication noncompliance. E.L. recalled having an “aura” for the first time on February 2014, seven months after his diagnosis of “epilepsy”. The symptoms (visual and auditory), were bilateral, described as “muffled” hearing and visual acuity deficits. His seizures have been controlled with oxcarbazepine monotherapy, an antiepileptic drug (AED) (300 mg at 8-hr intervals).

E.L. denies any prior history of head injury or trauma. He reports no other illnesses, and takes no other medications, apart from oxcarbazepine.

Social history is unremarkable. He does not smoke tobacco, denies excessive alcohol use, and denies use of recreational drugs.

E.L.’s family history is equally unremarkable. His parents are healthy and he has 3 brothers who are also alive and well. There are no known hereditary illnesses in the family. Cranial nerve testing showed decreased olfaction of vanilla on the right nostril, and normal on the left. Visual fields were full to confrontation testing. Fundoscopy was normal. Visual acuity was normal. Slight anisocoria was present (left pupil). Pupils were reactive to light bilaterally, and there was no afferent pupillary defect. Extraocular movements are full and symmetrical, but sustained extreme left gaze was impaired, and required frequent corrective saccades. Clear nystagmus was not observed. The remainder of the cranial nerves were unremarkable.

Motor exam showed symmetrical strength, bulk, and tone bilaterally. Reflex examination showed normal and symmetrical deep tendon reflexes for the most part except for slightly brisk brachioradialis on the left and the presence of a Trömner sign on the left. Plantar stimulation was unremarkable on the right, and elicited intense and persistent flexion on the left suggesting a grasp reflex. Other frontal release signs, including palmomental reflex, were absent. Sensation is intact throughout. Coordination testing showed symmetrical movements without dysmetria or incoordination on finger to nose testing and on sequential finger tapping during unimanual movements with either the left or right hand. *However, there is incoordination of sequential finger tapping movements in the left hand during bimanual movements (see main text).* In the beginning, E.L. expressed surprise that his left fingers become paralyzed for a few sec during the neurological examination (**Supplementary** **Movie 1**). Amazed, he remained opening and closing his empty left hand with grasping movements for 3-4 seconds. Then, E.L. pressed his palm flat against his left leg during 2-4 sec (**Supplementary Movie 1**). The recurrence of the temporary paralysis of the left-hand fingers, during subsequent neurological examinations, was not matter of surprise, but, of anger and frustration because of the feeling that he was unable to perform the controlled sequence during bimanual tasks. Balance is intact, even when tested with feet in tandem position.

**qEEG signals related to left/right hands and foot movements.**

Starting with the left hand alone, there were no significant differences in the frequency of finger-to-thumb events between E.L and the CTRL. But, E.L. with this experimental paradigm showed decrease in frequency of delta waves from 73.7 ± 0.4 to 56.6 ± 2.2 (µV)/epoch(n=3) (*P* <0.05)**,**but not for alpha, beta and theta (**Fig. 4**). CTRLs (n=5) showed no difference in all frequency bands between motor tasks executed with their left hand and right hand alone, at rest and during execution of finger-tapping.

**EEG spectral gradient (APSG)**

A lateralized antero-to-posterior EEG spectral gradient (APSG), which had greater low frequency power from the right prefrontal, frontal lobe and occipital areas, emerged as the most prominent feature at rest associated with E.L.’s brain lesion (**Fig. 4**). The APSG, characterized by delta and theta frequency rhythms, crossed over the anterior and posterior segments of the *C.C.* to reach the contralateral hemisphere. Frequency-related measures at the electrode site Fp2 (right PFC), under resting conditions, was higher for delta and theta waves (estimated frequency 74.2 ± 1.1, and 69.3 ± 3.8 (µV)/epoch (n=5), respectively) than for alpha and beta (estimated frequency 61.2 ± 3.1 and 46.2 ± 2.9 (µV)/epoch (n=5), respectively) compared to those in matched male CTRL subjects (n=16) (**Fig. 2g;** **Fig. 4**). Likewise, seizure-propagation patterns based on clinical observation suggested a similar spatial relationship between zones of seizure onset on E.L. primary sensorimotor cortex of the right hemisphere and propagation serving different motor functions within the homunculus, spreading from one point to another prior to becoming generalized. It is notable that other groups using fMRI achieved equal activation levels within identical anatomical localizations in CTRL for passive and active motor tasks with hand and foot movement paradigms^2^.

**Neuropsychological assessment**

Targeting domains of cognitive function and networks in which the PFC and the anterior *C.C*. are engaged, helped us select neuropsychological batteries to assess anticipated cognitive degradation, executive dysfunction, impaired attention and memory retrieval deficits. Since E.L.’s executive function and behavior did not deteriorate since his accident in 2012, we targeted the fMRI and EEG assays to document E.L. symptoms in detail, coupled with lesion mapping and analysis in an attempt to unveil functional compensation. E.L. did not present problems in family, sexual, social or professional life; he returned to work, had another child and no apparent cognitive or behavioral dysfunction. His performance-based standard test performance matched that of CTRL on a variety of memory, intelligence, attention and executive function, visuospatial, mood and behavior and motor function tests. E.L. did not present impairments with remembering facts, emotional memory, or recognizing previously seen faces and words. In agreement, subjects with right hemisphere injury show relatively intact use of grammar and vocabulary^3^. However, these patients manifest deficits in processing larger language units, such as deficit in expressing a coherent central meaning from text, as well as deficits in inferring the emotional content of discourse^3^. E.L. has not only been able to identify and explain ambiguous sentences, but also has been able to express himself with empathy, as illustrated in **Fig. 2f** “*I love my family and my parents*”. In addition, E.L. got married on 10/2019 and has not manifested depressive episodes since his seminal accident. On the contrary, his work-accident (2012), widely publicized, improved his mood and self-esteem. Accordingly, his epileptic episodes were due to medication noncompliance, quoted as *‘because (he) was* *feeling dangerously well’* (‘O. Sacks’ – to explain the paradox of E.L. feeling ‘too well’)^4^.

**Assessment of spontaneous social interaction**

As an alternative to standard neuropsychological test measures, direct observation within the natural setting, when possible, provides the most ecologically valid data for behavioral assessment^5^. Again, E.L. exceeded our expectations in every possible aspect. As an example, E.L. spontaneously helped a ‘granny cross the street’, carrying her luggage, in a real-life situation in Manhattan at the time of his assessment at NYU. He spontaneously made himself available and understandable because ‘she needed help’. With warmth in her face, she thanked him. They understood each other, even though they did not speak the same language and had different cultural backgrounds. Similarly, Gage did not speak Spanish, but he drove coaches, interacting with the public, and cared for horses in Valparaiso, Chile, conceiving a great fondness for animals and children^1^.

Living on unemployment benefits and earnings as a handyman, E.L. also shares household responsibilities with his wife, caring for his children and his home. The couple had another child on 01/17/2014 and married on 10/19/2019. In short, other than seizures, no findings following damage to E.L’s right PFC revealed disruption of his daily life, including personality, emotion and cognitive function (**Table 1; Extended Table 1**).

**Gage vs E.L.: uncovering evidence and evidence-based medicine**

In the absence of an autopsy and long-term assessment, each generation interprets Phineas Gage’s case anew in their own *patient*image. However, clinical reasoning and modelling on anatomical constraints support that Gage underwent a left transfixing frontal lobe injury^6^, uncovering the possibility of ‘recovery of function’, not observed in subjects with bilateral injuries^7^. Consistent with this view, Bigelow (1850) and Trevitt (1857)^1^ reported that ‘Gage recovered in faculties of body and mind, with only inconsiderable disturbance of function’^1^, paralleling recovery from encephalitis and seizures, in a pre-antibiotic and pre-anticonvulsant era.

The similarities are uncanny; although 164 years apart, the victims are of similar age, job, and suffered paralleled transfixing TBI. Gage´s ‘tamping iron’ (~6 kg) and E.L.´s ‘steel bar’ (4 kg), both low-energy high-speed transfixing projectiles, caused extensive left/bottom-up and right/top-down damage localized to frontal cortical regions and underlying WM, respectively^6^. Furthermore, neither victim lost consciousness and the severity of E.L. brain injury was remarkably similar and mirrored that of Gage’s, the latest modelled through population averaging^6^. While ~4% of the cortex was intersected in both cases by the bar´s passage, 11%-15% of total brain volumes were damaged, matching estimations of ~4% GM and WM damage in the PFC and pre-motor areas^6^. It follows from this perspective that interruption of WM tracts, either intrahemispheric (association fibers) or interhemispheric (commissural) fibers, could produce a variety of disconnection syndromes^8^, as they link cortical and subcortical gray matter into neural networks. In this study we identified injury in 3 WM tracts, also reported in Gage’s case^6^: superior longitudinal fasciculus, involved in core processes such as attention, memory and emotions; superior fronto-occipital fasciculus, involved in spatial awareness and symmetrical processing; and, cingulum fasciculus, projecting pathways that support core skills and executive functions (**Fig. 1i,j, Extended Data Fig. 5**), such as working memory, decision making, problem solving, and mental flexibility. In common, both the current study and previous modelling work on Gage’s skull^6^ and its effect on WM structure do not support the involvement of the anterior part of the *C.C*., involved in integration and transfer of interhemispheric information to process sensory, motor and high-level cognitive signals. It is notable that deficits in high-level cognitive control have been reported in patients with damaged longitudinal tracts, and that compromised cingulum fasciculus has been associated with memory disorders and mild cognitive impairment early in Alzheimer Disease^9,10^. Although damage to the connections between Gage’s posterior and anterior frontal regions are believed to account for his deficits in high-level cognitive control, E.L.’s network dysfunction depended on multitask demand. This consideration was emphasized by earlier work with patients with vascular malformations of anterior *C.C.*, unveiling emotional, behavioral, perception and spatial activity dysfunction of the right hemisphere^11^ . It turned out that E.L.’s cortical and subcortical lesions were not crucial contributions to his clinical presentation, with cognitive skills and behavioral performance similar to that observed in CTRL. Because experimental evidence supports prevalence of cognitive degradation, attentional dysfunction, and memory retrieval impairment in dementia patients with ≥ 25% of WM affected^12^, one may speculate that a lesser degree of compromise of E.L.’s WM obscured the appearance of signs and contributed to failure of clinical detection. As in previous investigations with subjects with callosal lesions, a wide range of WM involvement is needed to diagnose executive and behavioral dysfunction. However, some uncertainty arises because many patients with *C.C.* diseases have few, if any, obvious neurologic deficit^13^.

**Supplementary References**

1 Macmillan M, Lena ML. Rehabilitating Phineas Gage. *Neuropsychol Rehabil* 2010; **20**: 641–58.

2 Blatow M, Reinhardt J, Riffel K, Nennig E, Wengenroth M, Stippich C. Clinical Functional MRI of Sensorimotor Cortex Using Passive Motor and Sensory Stimulation at 3 Tesla. *J Magn Reson Imaging* 2011; **34**: 429–37.

3 Brownell H, Potter HH, Michelow D. Sensitivity to Lexical Denotation and Connotation in Brain- Damaged Patients: A Double Dissociation ? *Brain Lang* 1984; : 253–65.

4 Sacks O. Migraine. London, England, 2011.

5 Marcotte T, Grant I. Neuropsychology and the Prediction of Everyday Functioning. In: Neuropsychology of everyday functioning. 2010: 5–38.

6 Horn JD Van, Irimia A, Torgerson CM, Chambers MC, Kikinis R, Toga AW. Mapping Connectivity Damage in the Case of Phineas Gage. *PLoS One* 2012; **7**. DOI:10.1371/journal.pone.0037454.

7 Luria AR. Higher Cortical Functions in Man. 1966.

8 Catani M, Dominic H. The rises and falls of disconnection syndromes. *Brain* 2005; : 2224–39.

9 Ramsey LE, Siegel JS, Lang CE, Strube M, Shulman GL, Corbetta M. Behavioural clusters and predictors of performance during recovery from stroke. *Nat Hum Behav* 2017; **1**: 1–10.

10 Bozzali M, Giulietti G, Basile B, *et al.* Damage to the Cingulum Contributes to Alzheimer’s Disease Pathophysiology by Deafferentation Mechanism. *Hum Brain Mapp* 2012; : 1295–308.

11 Buklina SB. The Corpus Callosum, Interhemisphere Interactions, and the Function of the Right Hemisphere of the Brain. *Neurosci Behav Physiol* 2005; **35**: 473–80.

12 Filley CM. White Matter: Beyond Focal Disconnection. *Neurol Clin* 2011; **29**: 81–97.

13 Gazzaniga MS, Miller MB. The left hemisphere does not miss the right hemisphere. In: The Neurology of Consciousness. 2009: 261–70.
