# Supplementary material for: E.L., a modern-day Phineas Gage: Revisiting frontal lobe injury
Source: Lancet Reg Health Am. 2022 Aug 11;14:100340. doi: 10.1016/j.lana.2022.100340 (PMC9903712; doi:10.1016/j.lana.2022.100340)
Supplement: Supplementary file 16 — Supplementary Methods. [file mmc16.docx]

**Supplementary Methods**

**1) MRI**.

E.L. brain damage was examined following a modified template proposed by Damasio and Damasio (1989)^1^. The MR exam was performed in a 3T clinical scanner (Magnetom Verio, Siemens, Erlangen, Germany) by using a 12-channel head coil. T1-weighted images were acquired in the sagittal plane with a 3D magnetization prepared rapid acquisition gradient echo sequence (MPRAGE). The following parameters were used: 2530 msec repetition time (TR), 3.43 msec echo time (TE), 7 flip angle, 1100 msec inversion time (TI), 190 Hz/pixel bandwidth, 256 x 192 in-plane resolution, 1.3 mm slice thickness, 128 slices resulting in a voxel-size of 1 x 1 x 1.3 mm, no parallel imaging, one average, and a total acquisition time of 8:07 min. The image acquisition protocol was adapted to optimize the gray and white matter contrast. Sagittal T2-weighted Space 3D (TR/TE = 2800/489 ms, 176 slices resulting in a voxel size 1x1x1mm, 256x256 in plane resolution, no parallel imaging, one average, and a total acquisition time of 2:45 min), sagittal Fluid attenuated inversion recovery (FLAIR) 3D sequence (TR/TE = 5000/323 msec, 176 slices resulting in a voxel size 1x1x1 mm, 256x256 in plane resolution, no parallel imaging, one average, and a total acquisition time of 6:02 min) were included in the protocol.

***Single voxel MR-spectroscopy*** were performed using single and multivoxel techniques with PRES (point resolved spectroscopy) sequence. Single voxel technique was performed with the following parameters TR-1500ms; TE – 30 msec; Averages: 100; Volume - 20x20x20; Flip Angle - 90°; Prep Scan - 4; Bandwidth - 1200 Hz; Delta Frequency - -2.3 ppm. The multivoxel technique was performed with the following parameters TR – 1500 msec; TE – 30 msec; Averages - 2; Volume - 80x80 R>L e A>P; Thickness - 20 mm; FOV - 160x160 R>l e A>P; Flip Angle - 90°; Prep Scan - 4; Bandwidth - 1200 Hz; Delta Frequency - -2.7 ppm. Axial GRE EPI diffusion-weighted sequence (20 directions, TR/TE = 3200/104 msec, one average, b0/ b1 = 0/1000), 1562 Hz/pixel bandwidth, 128 x 128 in-plane resolution, 5 mm slice thickness, 128 slices were also obtained.

***Image Postprocessing and Volume Measurements****.* ***General Aspects****.* The T1-weighted images were transferred to a workstation (Mac Pro, 8 GB, 2 3.2 GHz Quad-Core Intel Xeon, Apple, USA), and volumetric analysis was performed using two automated (FreeSurfer and FSL tools) methods. The segmented 3D images were inspected for gross errors through visualization with 3D slicer (Version 3.2 1.0, NIH, USA), and volume values were extracted by implemented Unix scripts in the case of the automated methods. Within the scripts, it was possible to access the volumes of the regions analyzed of all processed volumes in a practical and fast manner. The intracranial volume (ICV), including white matter, gray matter, and cerebrospinal fluid, was also estimated using FS.

***Automatic Segmentation with FreeSurfer****.* Automatic subcortical volumetric segmentation was performed using the FS image analysis suite (v4.0.5 Martinos Center for Biomedical Imaging, Harvard-MIT, Boston, MA), which is documented and freely available for download. The technical details of these procedures can be described briefly. This fully automated process includes motion correction, removal of nonbrain tissue, automated Talairach transformation, segmentation of the subcortical white matter and deep gray-matter volumetric structures, intensity normalization, and cortical reconstruction. This segmentation procedure assigns a neuroanatomical label to every voxel in the MR image volume. The method is based on probabilistic information estimated from a manually labeled training set. The Markov Random Field Theory is applied, where the probability of a label at a given voxel is computed not just in terms of the gray-scale intensities and prior probabilities at that voxel, but also as a function of the labels in a neighborhood around the voxel in question. The time of the whole post-processing step took approximately 20 hr.

***Automatic Segmentation with FSL tools****.* Automatic segmentation of the subcortical structures was performed with FIRST (v1.2) (FMRIB Integrated Registration and Segmentation Tool, Oxford University, Oxford UK) within the FSL software package (v.4.1.0, FMRIB Analysis Group, Oxford, UK; FIRST is a model-based segmentation tool. The shape/appearance models used in FIRST are constructed from manually segmented subjects provided by the Center for morphometric Analysis (CMA), Massachusetts General Hospital MGH, Boston. The manual labels are parameterized as surface meshes from which a point distribution model is constructed. Based on these models, FIRST searches for the most probable shape among linear combinations of variations of shape models, given the observed intensities in the T1-weighted images of the study. The finite transformation to standard space is found, the subcortical structures are segmented, and a segmentation image of the hippocampus is produced. To avoid wrong results due to gross errors in registration to the standard space, each registration must be checked through an overlay to the MNI152 space image and, if necessary, the subcortical segmentation protocol must be re-run. Finally, a boundary correction of the segmented structures is applied that determines the voxels that are included or not included in a specific structure, using a z-threshold of 3. Volume measurements were extracted through a UNIX script. The entire post-processing procedure took approximately 20 min.

***Dynamic susceptibility contrast-enhanced (DSC) perfusion MRI.*** Before DSC perfusion MR imaging, a preloading dose of gadopentetate dimeglumine (Magnevist, 0.06 mmol/kg of body weight; Bayer-Schering, Berlin, Germany) was injected before DSC scan acquisition to correct for T1-weighted leakage effects. DSC perfusion MR imaging was performed during the injection of a bolus of gadopentetate dimeglumine (Magnevist, 0.07 mmol/kg body weight) with a dose of 0.1 mmol/kg (0.2 ml/kg) body weight at a rate of 5 mL/s through an intravenous catheter by using an MR-compatible pump (Medrad, Indianola, Pa); The bolus of contrast material was immediately followed by a 15-mL bolus injection of saline that was administered at the same injection rate. DSC perfusion MR imaging was acquired with a single-shot gradient-echo echo-planar imaging sequence (TR/TE, 1400/32 ms; flip angle, 60°; FOV, 23 cm; matrix, 128 × 128; section thickness/gap, 5/1 mm; in-plane resolution, no parallel imaging, one average, 1.8 x 1.8 mm; acquisition time, 2 min 45 sec). A total of 50 dynamic series of 19 sections to cover the entire brain were obtained. Subsequently, a contrast-enhanced T1-weighted sequence was performed. To analyze the DSC perfusion MR imaging data, we used commercially available built-in software (Siemens). Perfusion maps of relative cerebral blood volume (rCBV) was generated. We used a gamma-variate fit to derive hemodynamic parameters from the DSC MR image to obtain the map of relative CBV, in arbitrary units. After eliminating the recirculation of contrast agent with gamma-variate curve fitting, we computed the relative CBV with a numeric integration of the curve. The shape of the arterial input function was determined from the proximal middle cerebral artery contralateral to the affected hemisphere.

***Intracranial Volume*.**The ICV was estimated automatically within the FS processing pipeline using the same T1-weighted images.

***Diffusion-Tensor MR Imaging*.**In the DT-MR imaging protocol, 4 sets of axial single-shot spin-echo echo-planar (EP) images (*b* = 0 and 1000 s/mm2) were collected with diffusion gradients applied sequentially along 12 noncollinear directions. Five acquisitions consisting of a baseline T2-weighted echo-planar (EP) image and 6 diffusion-weighted EP images, a total of 35 EP images, were collected per section position. The acquisition parameters for the EP imaging sequence were 15 axial sections of 5-mm thickness and 1.0-mm section gap, a FOV of 240 × 240 mm, an acquisition matrix of 128 × 128 (zero filled to 256 × 256), a repetition time (TR) of 10 sec, and an echo time (TE) of 98.8 msec, acceleration factor (SENSE) of 2, and the scanning time for DTI acquisition of 3 min 40 sec. A two-seed point procedure was used for fiber tractography. For each of the two fiber structures, two ROIs served as end points: one cortical and another in the ipsilateral brain stem.

***Functional MR Imaging (fMRI)*.**The functional data were acquired using the blood oxygen level–dependent BOLD technique. Contiguous multisection gradient-echo echo-planar imaging was used, with parameters of 2000/60 msec (TR/TE), 14 sections, 64 × 64 matrix, and a section thickness of 5 mm with no gap. We used a block motor finger opponent movement paradigm with 20 sec for the task period and 40 sec for rest. The task consisted of 90 images for 6 cycles. The patient's compliance with fMRI paradigms, functional brain activity, and head motion were monitored in real-time by using available software. The raw functional MR imaging data were analyzed off-line using an independent workstation (Leonardo, Siemens). Functional MR imaging maps were generated using a cross-correlation technique. The functional MRI data was co-registered to the high-resolution contrast-enhanced image obtained before and with the tractography. Images were acquired during hand and feet movement paradigms. The difference between **active** and **passive** range of **motion** is that the latter was carried out by a lab assistant.

**2) 3D printed brain models.** A 3D model of E.L.’s brain was created from overlapping image layers from the MRI datasets (see MRI - Methods) using the software Mimics (Materialize v. 14, Leuven, Belgium). Segmented images, converted into STL format, were exported to a SLS printer (EOS P110, Electro Optical Systems, Germany) as previously described^2^.

**3) Quantitative Electroencephalography (qEEG)**. A TiEEG1 (EMSA) was used to amplify and record EEG data from 23 scalp sites with tin electrodes fitted in the 10-20 international system. The electrode impedances were always <5 kOhms at all sites and the maximum of frequency filter was 70 Hz, with a Notch filter in 60 Hz. It was used a sampling of 200 p.c.s., a voltage gain of 100 µV/cm and a recording of 22 min and 15 sec. E.L. sat in a comfortable chair in a dimly lit room and completed the inventories while two of the investigators attached the electrodes. He was instructed to move as little as possible and to avoid eye movement during EEG recording. The brain mapping was made based on the histograms of absolute amplitude produced by the EEG. The power on frequency bands (delta 0.5 - 4.0 Hz, theta 4.0 - 7.9 Hz, alpha 8.0 - 12.5 Hz, beta1 12.6-19.9 Hz) were represented as color disposed on a stylized picture of the brain.

**4) Eletrooculography (EOG)**

**Saccade & Antisaccade Task.** The patient and 5 male subjects with age between 20 and 30 years old (CTRL) were tested in a silent room. The fixation point was of 0.5° of visual angle and the target was of 2.0°. They were presented in the horizontal plane at eye level. A 20” monitor was used, located 57.3 cm from the subject. Tasks were administered to all subjects randomized, with the instructions of presenting a prosaccade (looking at the same direction of the stimulus) or an antisaccade (looking at the opposite direction of the stimulus), being displayed in the monitor before the appearing of the central fixation point. An examiner provided instructions before the task and to facilitate the comprehension, the target had the same color of the instructions displayed in the monitor (saccade–green, antisaccade-red).

***Overlap task.*** After the display of the instructions for 2.3 sec, appears the central fixation point for 0.5 – 1.5 sec in a random way, which the subject is instructed to look at as long as the target does not appear. Then, with a 20° distance (right or left) from the central fixation point, appears a square target of 2.0° for 1.0 sec, to where the subject must fix his gaze.

***Gap task***. This task has the difference of central fixation point vanishing before the appearance of the target, with a gap of 0.2 sec. Each task has 20 antisaccades and 20 saccades, 10 for each side. Each task was made 2x with the subjects and 3x with the patient. Eye movements were measured by electrooculography (EOG); using two electrodes placed near the external canthus of each eye, in the horizontal plane across both eyes (Neuron-spectrum 5 - Neurosoft). It was also used an AC EOG amplifier with filters set to band-pass 0 to 15 Hz. Data were digitized via cable with the use of the software Neuro-spectrum.net. The analysis of oculomotor recordings was performed using in-house developed software with MatLab-R2013b, to identify the types of responses, errors, and the latencies of the responses.

***Oxcarbazepine test*.** Saccade and Antisaccade tasks were used in 5 CTRL subjects (randomly selected out of the original CTRL pool of 10 subjects; age between 21 and 25 years old) under the influence of Oxcarbazepine. It was administrated 300 mg of Oxcarbazepine 3x/day (900mg of daily dosage) for 48 hr, to match the plasmatic concentrations of E.L. After this period, the CTRL was submitted to the Saccade and Antissacade protocol, performing 3 Overlap tasks and 3 Gap tasks each (6 tasks/subject).

The subjects and the patient´s body mass composition and fat/lean mass correlation were measured through InBody 270 BridgePower Corp.  (Model BPM04OS12F07) Direct Segmental Multi-frequency Bioelectrical Analysis Method (DSM-BIA). Impedance was applied by using 2 different electrical current (200 µA) at each of 5 body segments (Upper and lower limbs and trunk) contacting the tetrapolar 8-point tactile electrodes, lasting 3 sec per measurement. Current latencies were registered and correlated to the subject's body composition, considering height, weight, gender, and age.

***Statistical analysis.*** Comparisons were realized between E.L., selected CTRL group 21.6 ± 0.9 (20-30 years) (n=5) without oxcarbazepine influence and under oxcarbazepine influence (CTRL OXC – n=5). Calculations for each paradigm were performed with the open source software Singlims ES using the Crawford and Howell's and the Crawford & Garthwaite´s methods^3^, supplemented by point and interval estimates of effect sizes tests if E.L’s mean score was significantly below CTRL. This methodology provides a point estimate and percentage of the abnormality of the score, with the use of *P* value, and sets confidence limits on the abnormality of a patient’s score using non-central t-distributions. A *P* value <0.05 under one-tail probability were considered statistically significant.

The nonoverlap of all pairs (NAP) method compares every point in one phase with every point in the succeeding phase. NAP is a nonparametric technique for measuring the percent of nonoverlapping data between baseline (A phase) and treatment phases (B phase). NAP is calculated as the proportion of all pairs of one observation from each phase in which the measurement from the B phase improves upon the measurement from the A phase. The range of NAP is [0,1], with a null value of 0.5.

**5) Neuropsychological testing**

**Neuropsychological and assessment of sexuality following TBI (BIQS)**. E.L. participated in a comprehensive assessment including assessment of cognition, achievement, language, memory, executive function, attention, and behavioral/emotional status in a clinic setting. A neurologist and a licensed psychologist independently administered and reviewed all neuropsychological measures consistent with standardization. The measures were administered in a random order; however, continuous performance tests were counterbalanced. E.L. continued with the AED medication as prescribed, without interruption. E.L. and his spouse independently completed the *Brain Injury Questionnaire of Sexuality* (BIQS), placed it in an envelope and an independent nurse sealed the envelope to ensure confidentiality^4^.

**Dual task test.** Paper-and-pencil type dual-task (Oiso-DT), for assessing inattention of brain-damaged patients (Minoru Toyokura)^5^, corrected for subjects with low level of education (i.e., incomplete Junior High School). In the test, the subject performs a simple cancellation task simultaneously with a writing calculation (addition) task. In the cancellation task, an audio device plays aloud the numbers 1 to 19 in a random order, and the subject replies “Yes” whenever the target number “8” is presented. A total of 90 numbers are played at intervals of 2 sec and a total of 20 “8s” are played. In the calcula­tion task, the subject must mentally calculate and write as many figures of 2 digits as possible. The subjects were instructed not to skip any calculation task that they did not know and were not allowed to return to top page to double check their answers. The test was applied twice, beginning with equations structured with horizontal lines and then, with vertical lines. The cancellation task was scored based on the cor­rect response rate (correct answer/20) (%) and the calculation task was scored based on the number of tasks computed, number of correct answers, and the rate of correct answers (the number of correct answers/number of tasks computed). The performance of CTRL was ob­tained from the results of 10 male volunteers, 33.4 ± 2.4 (20-45 years) with incomplete junior high school, to match E.L. level of education.

**Iowa Gambling Task (IGT).** The IGT was designed to assess decision-making abilities^6,7^. Participants are given a fictitious amount of $2K and instructed to maximize winnings while choosing repeatedly from four decks (A-D) of playing cards that unpredictably yield wins and losses. Importantly, the contingencies of reward and punishment are counter-intuitively arranged so that the decks A, B with higher wins ($100) result in a long-term net loss, while the decks C, D with smaller wins ($50) yield a net gain. Participants who do not learn to prefer one or both of the $50 decks over the course of 100 trials are considered to exhibit a decision-making impairment, critical to good IGT performance. The mean net score is derived from the number of choices from good decks minus number of choices from bad decks.

**Apathy Evaluation**

***Apathy Evaluation Scale - Clinician Version (AES-C).*** Both E.L. and CTRL 26.7 ± 2.7 (20-35 years) (n=4) were assessed one week prior to the TMS stimulation. This validate method is employed as a guided interview by a physician to subject in order to screen, quantify and characterize apathy considering the past 3 weeks of the subject´s activities, behavior and routine ^8,9^.

***Scale for the assessment of negative symptoms (SANS).*** Both E.L. and CTRL 26.7 ± 2.7 (20-35 years) (n=4) were assessed one week prior and 10min after cTBS. The scale consists of five subscales that evaluate five different aspects of negative symptoms: alogia, affective blunting, avolition-apathy, anhedonia-asociality, and attentional impairment. This validated method is used to assess the presence of negative symptoms and its changes over time^10,11^.

***Logical Memory subtest of the Wechsler Memory Scale III (WMS III).*** This subtest of the WMS III is composed of two stories (A and B) with 25 items each. The investigator reads one story each time, and the subject is asked to recall each one immediately after the presentation. The second story is repeated once. A delayed recall is done 30 min after the presentation. The score is the number of items correctly recalled^12^.

***Digit span subtest of the Wechsler Memory Scale - Revised (WMS - R).*** Subjects were asked to repeat in the same order (forward) and in the reverse order (backward) a series of digits recited orally by the examiner. The forward test evaluates verbal short-term memory (the phonological loop), and the backward test evaluates the ability to maintain and manipulate sequences (central executive function)^12^.

***Phonological verbal fluency (P) and semantic verbal fluency.*** The subject is asked to produce spontaneously as many words beginning with the letter “P” as possible within a 2-min interval, provided that proper names, repetitions of the same word, and derivations are not used. This test evaluates verbal fluency under conditions that require an active search of words in the lexicon and inhibitory control (executive function) to prevent unwanted words. The score is the total number of words given by the subject. In the animal category test, the subject is asked to produce as many animal names as possible within 2 min. This test evaluates semantic verbal fluency that is dependent on the functioning of semantic networks and is used as a measure of semantic memory^12^.

***Corsi block-tapping task.*** This task is used to measure visuospatial short-term memory, The experimenter taps wooden blocks in a random sequence, and the participant is asked to reproduce the block tapping^12,13^.

***Trail Making A and B.*** This test of complex visual scanning is a classic executive task. It evaluates the speed of visual search and requires mental flexibility, attentional resources, and motor abilities. Both parts require perceptual tracking of a sequence and fast performance, but part B also requires divided attention. Applications are preceded by a training session, in which the errors are pointed out and should be corrected by the testing. The test ends after three errors or 5 min after start of the task. The score is the time taken to ´perform the task^12^.

***Raven Progressive Matrices (PM 38).*** In this test of general intelligence, the subject is presented with five series of 12 different pictures that contain a small segment to be completed. The score reflects the number of segments correctly identified^12^.

***Tower of London.*** This fundamentally evaluates planning and problem-solving capacity. It is an assessment of the ability to maintain actively goals and other task-relevant information in distracting and conflicting contexts. Participants are required to move colored beads from an initial state to a goal state. They need to perform mental planning before they execute the task to reach the goal, following specific predetermined rules^12^.

***The Stroop Color Word test.*** Its main objective is the evaluation of one crucial aspect of executive functions: control of interference^12^. Patient is required to read three different tables as fast as possible. The first two of them represent the “congruous condition” in which participants are required to read names of colors printed in black ink (Stroop-W) and name different color patches (Stroop-C). Conversely, in the third table, named color-word condition (Stroop-CW), colored words are printed in an inconsistent color ink (for instance the word “red” is printed in blue ink) and the patient is requested to say the color of the ink instead of the word. Golden and Freshwater(1978)’s scoring method was adopted for this essay^14^.

***Wisconsin Card Sorting Test.*** This test measures the ability to form, maintain, and shift cognitive set and to inhibit a prepotent response. It evaluates abstract reasoning and cognitive flexibility. The test was administered using the Nelson version^15^.

***California Verbal Learning Test.*** It is used to assess explicit verbal memory. Total number of words initially recalled over the first five trials, delayed recall of the original list and recognition was measured. Learning test with a list of 16 words divided into 4 semantic categories. List A is presented orally, 5 times. For each presentation, the individual must recall the totality of the words he remembers. At the end of the 5th presentation, a list B is presented only once, with 16 words divided into 4 semantic categories, and only two categories coincide with those of list A. At the end, list A must be evoked again and, later, after 20 min. A recognition step completes the test^16^.

***Grober and Bushke Test.*** This 16-item list-learning task allows for measurement of encoding with semantic clues, and short-and long-term storage and retrieval processes. For each evocation of the 16-word list, there is a first free evocation step followed by a second evocation step with clues for the items not evoked in the first step. Thus, we have the results corresponding to free evocations and in total (free + with clues)^17^.

***Boston Naming Test.*** It measures confrontation naming, the ability to pull out the correct word at will. The patient has to name 60 black and white line drawings of objects in familiarity. Number of correct responses correlates with the score^12^.

***DMS 48.*** This is an image recognition test. 48 images are presented one by one, and the individual memorizes them incidentally. During the acquisition phase, he does not know that it is a memory test, he only has to say if the image presented has more or less than three colors. At the end of this phase, an interval of approximately 3 min is filled by performing the phonemic verbal fluency test. At the end of this, we then move on to the test phase, where the target image, presented in the acquisition phase, is presented with another image, and the individual must say which of the two he saw during the acquisition phase. An answer is required even if the individual says he does not remember (forced choice paradigm). The 48 images are divided into three groups of 16 images: a “single” group, where each image, in the test phase, is presented with a distractor with no semantic or lexical link, a “paired” group, where each image is presented with a similar distractor in terms of shape, color and name, and an “abstract” group, where the targets and distractors are abstract stimuli, difficult to verbalize^12^.

***Ruche learning Test.*** Visuospatial memory test. 41 squares are displayed, 10 of which are represented in black. The subject must memorize them for 45 sec, and play them back immediately afterwards. There are 5 attempts, an immediate recognition step, and a delayed recall step after 10 min^12^.

***Beck Depression Inventory (BDI)***. A validated screening method for detecting depression in TBI patients^18^, it’s a 21-question multiple-choice self-report questionnaire for detecting depressive symptoms^19^.

**6)** **Transcranial Magnetic Stimulation (TMS).**

**Neuronavigation.** The following parameters were used: 2400 msec repetition time (TR), 3.67 Msec echo time (TE), 1.2 mm slice thickness, voxel-size of 1.2 x 1.2 x 1.2 mm and 192 total axial slices.

***EMG signals*** of the first dorsal interosseous muscle were recorded with surface EMG electrodes (Ag/AgCl electrodes, 2.0-cm interelectrode distance) adhered to the skin in a bipolar arrangement over the FDI muscle belly. For navigation, the BrainSight software system was used, for the visualization of the coil location in relation to the cortex. After co-registration, the image of the patient’s brain was represented in a coordinate system (defining coordinate X, Y, and Z), such that the position of the coil with respect to the brain could be visualized on the corresponding three-dimensional (3D) MR image shown by the navigation system.

Dorsolateral PFC (***dlPFC)*.** TMS was performed with the figure-of-8 coil, positioned tangentially to the convexity of the head above the left DLPFC. The target location was identified on the first day on which rTMS was administered and was based on the International 10/20 System for EEG and aided by a tool developed for left DLPFC identification^20^. Trains of rhythmic low-frequency (1 Hz) rTMS were delivered for 3 min during each session. A total of 180 pulses were applied. Stimulation intensity was set to 120% of each participant’s resting motor threshold (RMT). This procedure allowed us to start the ROFC Test 3 min (IR ROFC) after the cessation of rTMS train. Neuropsychological ROFC evaluations were performed at 3 time points: copy (prior to TMS-Treatment), 3 min (IR) and 30 min (DR) after rTMS.

**Left Primary Motor Cortex (left PMC).** We employed two regimens of rTMS protocols, one excitatory (10Hz) and the other inhibitory (1Hz) on different days in the left PMC with a Cool-B65, butterfly (figure 8) coil. The excitatory regimen consisted of 40 trains of 20 pulses at 10Hz (2 secs each), with an intertrain interval of 25 sec at 100% RMT. The inhibitory regimen consisted of 1200 pulses at 1Hz (total time 20 min) at 120% RMT. This protocol results in suppression of excitability of the targeted cortical region for 20-40 min following completion of the rTMS.

Ventromedial PFC ***(vmPFC)*.** We employed the continuous Theta-Burst Stimulation (cTBS) technique, lasting 40s, at 80% RMT. Marked individual scans were imported to a BrainSight 2 neuronavigation system (Rogue Research Inc, Montreal, Quebec), and each region of interest was targeted with the TMS coil Cool D-B80, Butterfly – preferred for deeper cortical structures as vmPFC – using its motion-capture tracking function. The vmPFC stimulation was performed at 0° from the midline, with the coil being placed tangentially to the forefront (i.e. almost vertically). A total of 200 burst trains were applied at a frequency of 5Hz, with 3 pulses/burst and a pulse frequency of 50Hz - giving a total amount of 600 pulses. This protocol results in suppression of excitability of the targeted cortical region for 40-60 min minhiur following completion of the rTMS. The rTMS parameters were well within currently recommended guidelines. EEG recordings were performed with a Nihon Kohden EEG 1200K instrument using 20 scalp electrodes

**Supplementary References**

1 Damasio H, Damasio AR. Lesion analysis in neuropsychology. New York: Oxford University Press, 1989.

2 Werner H, Santos JRL dos, Fontes R, *et al.* dditive manufacturing models of fetuses built from three-dimensioesonance imaging and computed tomography scan data. *Ultrasound Obs Gynecol* 2010; **36**: 355–61.

3 McIntosh RD, Rittmo J. Power calculations in single-case neuropsychology: A practical primer. *Cortex* 2021; **135**: 146–58.

4 Stolwyk RJ, Downing MG, Taffe J, Kreutzer JS, Zasler ND, Ponsford JL. Assessment of sexuality following traumatic brain injury: validation of the Brain Injury Questionnaire of Sexuality. *J Head Trauma Rehabil* 2013; **28**: 164–70.

5 Toyokura M, Nishimura Y, Akutsu I, Watanabe F. Clinical significance of an easy-to-use dual task for assessing inattention. *Disabil Rehabil* 2017; **39**: 503–10.

6 Bechara A, Damasio AR, Damasio H, Anderson SW. Insensitivity to future consequences following damage to human prefrontal cortex. *Cognition* 1994; **50**: 7–15.

7 Bechara A, Tranel D, Damasio H. Characterization of the decision-making deficit of patients with ventromedial prefrontal cortex lesions. *Brain* 2000; **123**: 2189–202.

8 Marin RS, Biedrzycki RC, Firinciogullari S. Reliability and validity of the Apathy Evaluation Scale. *Psychiatry Res* 1991; **38**: 143–62.

9 Clarke DE, Reekum R Van, Patel J, Simard M, Gomez E, Streiner DL. An appraisal of the psychometric properties of the Clinician version of the Apathy Evaluation Scale (AES-C). *Int J Methods Psychiatr Res* 2007; **16**: 97–110.

10 Rao V, Spiro JR, Schretlen DJ, Cascella NG. Apathy Syndrome After Traumatic Brain Injury. *Psychosomatics* 2007; **48**: 217–22.

11 Andreasen NC. Negative Symptoms in Schizophrenia. *Arch Gen Psychiatry* 1982; **39**: 84–788.

12 Strauss E, Spreen ESEMSSO, Strauss PPE, *et al.* A Compendium of Neuropsychological Tests: Administration, Norms, and Commentary. Oxford University Press, 2006 https://books.google.com.br/books?id=jQ7n4QVw7-0C.

13 Kessels RPC, Van Zandvoort MJE, Postma A, Kappelle LJ, De Haan EHF. The Corsi Block-Tapping Task: Standardization and normative data. *Appl Neuropsychol* 2000; **7**: 252–8.

14 Golden CJ, Freshwater SM. Stroop color and word test. 1978.

15 Nelson HE. A modified card sorting test sensitive to frontal lobe defects. *Cortex* 1976; **12**: 313–24.

16 Yi A. California Verbal Learning Test (California Verbal Learning Test-II) BT - Encyclopedia of Clinical Neuropsychology. In: Kreutzer JS, DeLuca J, Caplan B, eds. . New York, NY: Springer New York, 2011: 475–6.

17 Grober E, Buschke H, Crystal H, Bang S, Dresner R. Screening for dementia by memory testing. *Neurology* 1988; **38**: 900–3.

18 Green A, Felmingham K, Baguley IJ, Slewa-Younan S, Simpson S. The clinical utility of the Beck Depression Inventory after traumatic brain injury. *Brain Inj* 2001; **15**: 1021–8.

19 Beck AT, Ward CH, Mendelson M, Mock J, Erbaugh J. An inventory for measuring depression. *Arch Gen Psychiatry* 1961; **4**: 561–71.

20 Mir-Moghtadaei A, Caballero R, Fried P, *et al.* Concordance Between BeamF3 and MRI-neuronavigated Target Sites for Repetitive Transcranial Magnetic Stimulation of the Left Dorsolateral Prefrontal Cortex. *Brain Stimul* 2015; **8**: 965–73.
